# Expanding the genetic variation of *Brassica juncea* by introgression of the *Brassica rapa* genome

**DOI:** 10.1093/hr/uhab054

**Published:** 2022-01-19

**Authors:** Li Zhang, Xiangsheng Li, Lichun Chang, Tianpeng Wang, Jianli Liang, Runmao Lin, Jian Wu, Xiaowu Wang

**Affiliations:** Institute of Vegetables and Flowers, Chinese Academy of Agricultural Science, 100081 Beijing, China

## Abstract

*Brassica juncea* is an important vegetable and oil crop cultivated worldwide. To increase its genetic variation, we introgressed the A genome of *Brassica rapa* into *B. juncea*. We used three each of heading and semi-heading *B. juncea* accessions as recipient parents and a *B. rapa* line, B9008, as the donor parent. We obtained 101 BC_1_S_1_ lines in total with expanded phenotypic variations such as leafy head shapes. We developed 132 single-nucleotide polymorphism (SNP) markers that could distinguish the A genome of *B. juncea* from the *B. rapa* genome, and tracked the introgression of *B. rapa* segments in the new *B. juncea* germplasm. On average, 59.2% of the *B. juncea* A genome in the *B. juncea* introgression lines was covered by the donor segments. We also identified three markers whose donor genotype frequencies were significantly lower than the theoretical value, suggesting strong selection of the recipient genotype during the introgression process. We provide an effective strategy to evaluate the diversity of the new germplasm based on the combination of parental resequencing data and marker genotyping results. Further genetic analysis of 1642 SNPs showed that the genetic diversity of the new *B. juncea* germplasm with the introgressed *B. rapa* genome was significantly increased. This study illustrates the potential for expanding the genetic diversity of *B. juncea* through the introgression of the *B. rapa* genome.

## Introduction

There are six species of cultivated *Brassica*s, three of which are diploid species, *B. rapa* (AA, *n* = 10), *B. nigra* (BB, *n* = 8), and *B. oleracea* (CC, *n* = 9), and three of which are allotetraploid species, *B. juncea* (AABB, *n* = 18), *B. napus* (AACC, *n* = 19), and *B. carinata* (BBCC, *n* = 17). The three allotetraploid *Brassica* species were derived from the hybridization and polyploidization of two of the three diploid *Brassica* species. The relationship of *Brassica* species has been described using the ‘triangle of U’ model [1]. *B. juncea*, also known as mustard, has been widely cultivated as an important vegetable, condiment, and oilseed crop in China and some Southern Asian countries [2]. Long-term natural and human selection has resulted in many *B. juncea* subspecies that possess a variety of root, stem, leaf, and seed stalk forms [3, 4]. *B. juncea* var. *capitata* Hort (heading *B. juncea*) is a subspecies with a unique leafy head that is consumed as a fresh or pickled vegetable.

The leafy head is an important nutrient storage organ that contains substantial dietary fiber and vitamins [5]. In addition to heading *B. juncea*, a leafy head is also a characteristic trait of other *Brassica* species, including Chinese cabbage (*B. rapa* ssp. *pekinensis*) and cabbage (*B. oleracea* var. *capitata*). Even though a leafy head is a common trait for these three species, *B. juncea* shares its unique characteristic of leafy head structure with *B. rapa* and *B. oleracea*. The petiole of heading *B. juncea* is short and wide. As the plant grows, the central leaves overlap and wrap into the leafy head. The plants are often cracked because of the thickened petiole, and poorly developed heads have reduced the commercial value [6]. The formation and improvement of the leafy head have been the focus of many studies [7]. The genetic base of *B. juncea* is restricted by polyploidy, breeding activities, and a short evolutionary history [8–11]. The narrow genetic base has also limited the improvement and productivity of this crop [12]. It would be useful to enlarge the genetic base of *B. juncea* through introgression with a related species [13].

Introgression is the transfer of genes from one species to the gene pool of another by repeated hybridization [14, 15]. It is an important method for the transfer of favorable genes and to develop improved varieties. Introgression has been used for generating novel allotetraploid *Brassica* germplasm [16, 17], improving the yield of *B. napus* [18, 19], and expanding the genetic base of *B. carinata* [20]. Diploid progenitors can be used as genetic resources for the introgression of beneficial genes to improve allotetraploid plant traits [21]. Chinese cabbage is a commonly consumed vegetable in China and East Asia [22]. It has been selected for high yield and a compact leafy head [23]. Consequently, Chinese cabbage has great potential for improving the leafy head of *B. juncea*.

**Table 1:** Number of progenies obtained from donor parent B9008

Recipient parent and recurrent parent	Morphotype	No. of BC_1_S_1_ lines	No. of selected plants
20JS1535	Semi-heading I	13	14
20JS1539	Semi-heading I	19	20
20JS1537	Semi-heading II	41	44
20JS1536	Heading	3	3
20JS1538	Heading	16	17
20JS1540	Heading	9	9
Total		101	107

The identification of introgressed segments in hybrids is critical in molecular breeding. The widespread use of molecular markers has provided breeders with a powerful tool to precisely select the desired genotype [24]. Genetic introgression can also be assayed using molecular markers [25–27]. Single-nucleotide polymorphism (SNP), as a third-generation marker system with high polymorphism and high density across the genome, has been used for crops such as *B. rapa* [28] and *B. napus* [29]. Competitive allele-specific PCR (KASP) is currently the most popular SNP genotyping technology. It has high throughput and high accuracy, and can be used in marker-assisted selection [30]. However, KASP markers suitable for assisting large-scale interspecific introgression in Brassicaceae species have not been developed. In addition, the genetics of fragments introgressed from one of the contributor diploid species to the allotetraploid species is largely unknown.

**Table 2:** Number of A-genome-specific SNP markers on each chromosome of *B. rapa*

Chromosome name	Chromosome length (bp)	Available SNP markers	Average density (Mb)	Average genetic distance (cM)
A01	29 595 527	15	1.649	4.113
A02	31 442 979	12	2.643	7.878
A03	38 154 160	17	1.710	6.535
A04	21 928 416	10	2.062	6.590
A05	28 493 056	10	2.958	10.658
A06	29 167 992	14	2.004	6.807
A07	28 928 902	15	1.862	5.328
A08	22 981 702	10	2.160	7.367
A09	45 156 810	15	2.885	6.523
A10	20 725 693	14	1.481	5.440

**Table 3:** List of parental materials used in hybrid combinations

ID	Accession	Type
20BS1534	B9008	Self-compatible *B. rapa*
20JS1535	Si chuan da rou qing	Semi-heading I *B. juncea*
20JS1539	Ge li chao zhou bao xin jie	Semi-heading I *B. juncea*
20JS1537	Nan hai si ji hou rou da ban jie	Semi-heading II *B. juncea*
20JS1536	Shan dong ji xin jie	Heading *B. juncea*
20JS1538	Kuan bang bao xin qing cai	Heading *B. juncea*
20JS1540	Da rou bao xin jie	Heading *B. juncea*

*B. juncea* parents were divided into three categories according to the shape of the leafy head.

In this study, we describe the development of new *B. juncea* germplasm by introgressing the *B. rapa* genome (*B. juncea* introgression lines) to broaden the genetic variation of heading *B. juncea*. We developed a set of 132 polymorphic SNP markers that were used to distinguish the A genome of *B. juncea* from that of *B. rapa* based on resequencing data of the parents. We demonstrate *B. juncea* introgression lines carrying chromosome segments of *B. rapa* using newly developed KASP markers. The introgression breeding approach increased the morphological variation of the leafy head in *B. juncea*. Based on the genotyping results of KASP markers, the genomic polymorphisms of the *B. juncea* introgression lines were further evaluated, providing new insights into the diversity assessment of the new germplasm.

## Results

### Generation of *Brassica juncea* introgression lines

The initial interspecific crossing between *B. rapa* and *B. juncea* required embryo rescue to generate *F*_1_ hybrids. The authenticity of the hybrid combination (AAB) was verified by the low number of seeds per silique. Six true *F*_1_ plants were backcrossed to the recipient parents to generate a BC_1_ population. The surviving BC_1_ plants were self-pollinated and a total of 101 lines were produced (Table 1). Based on the phenotype and growth status of the derived lines in the field, we selected 107 plants for subsequent analysis.

### Morphological variation was expanded in the *Brassica juncea* introgression lines

**Figure 1:**
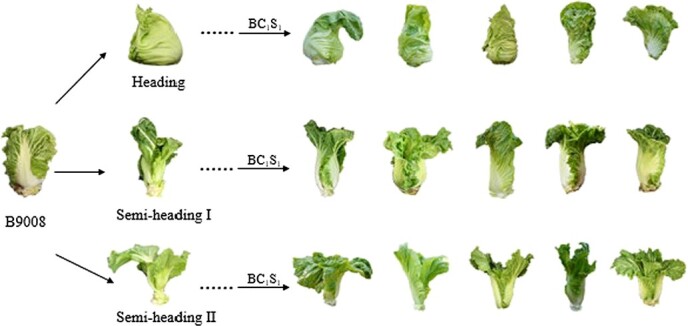
Typical phenotypes of 15 *B. juncea* introgression lines and their parents. Representative progenies of the BC_1_S_1_ population were obtained by crossing *B. rapa* accession B9008 with three categories of *B. juncea*.

We divided the *B. juncea* parents into three categories according to the shape of the leafy head. The leafy head of the heading category was approximately spherical. The other two categories were both semi-heading, and the petiole of semi-heading II was wider and thicker than semi-heading I. The progenies of heading *B. juncea* that had been introduced into the *B. rapa* genome retained the leafy head. In the progenies of semi-heading I, the formation of the leafy head has been improved, which was evident as the heading leaves around the shoot apexes continued to fold upward and inward to form a more compact head. The leaf shapes of the progenies generated by semi-heading II *B. juncea* changed more profoundly than those of leafy heads. Compared with the *B. juncea* accessions, the *B. juncea* introgression lines exhibited a wide range of phenotypic variations and showed similar characteristics to *B. rapa* (Fig. 1).

**Figure 2:**
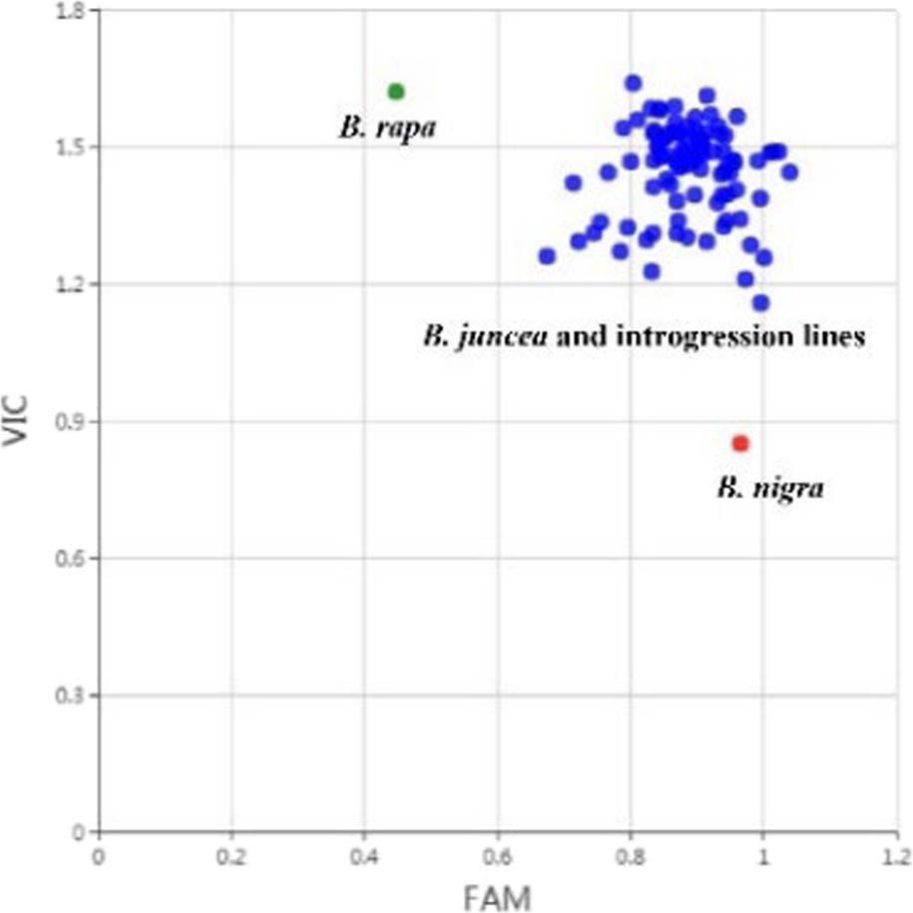
KASP genotyping for detection of the B genome with marker B01_40.

**Figure 3:**
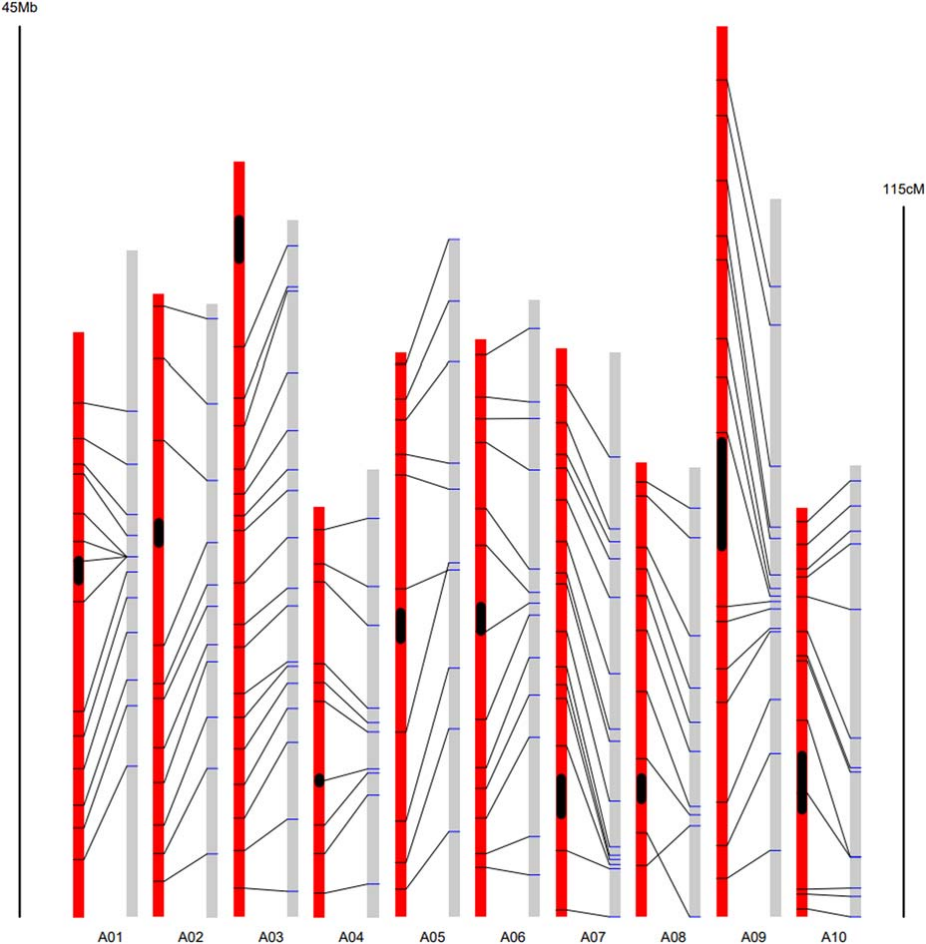
Physical location and corresponding genetic distance of SNP markers on the A genome. Red columns represent the 10 *B. rapa* chromosomes and gray columns represent the genetic linkage group corresponding to each chromosome. The black box indicates the centromere region.

**Figure 4:**
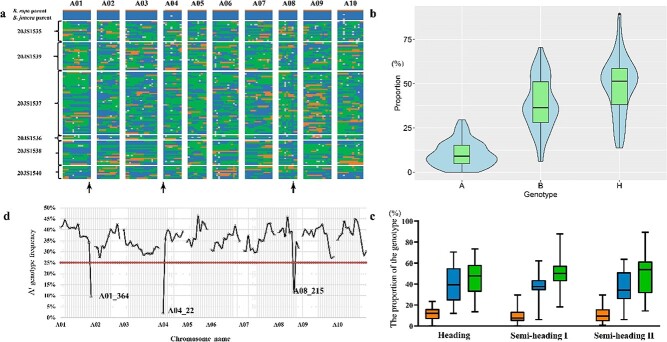
Genotyping results and analysis of 132 polymorphic markers in 107 *B. juncea* introgression lines. **a** Graphical genotyping based on polymorphism for BraA and BjuA SNP markers. The vertical axis represents parental materials and 107 *B. juncea* introgression lines, and the horizontal axis represents 132 SNP markers. *B. juncea* introgression lines were grouped by their corresponding *B. juncea* parents. The 132 SNP markers were arranged according to their physical positions on the chromosomes. Orange segments indicate the homozygous A^r^A^r^ genotype (A); blue segments indicate the homozygous A^j^A^j^ genotype (B); green segments indicate the heterozygous genotype (H); gray segments indicate the missing genotype (D). **b** Proportion of the *B. rapa* genome in the *B. juncea* introgression lines. Violin plots representing proportions of genotypes A, B, and H. **c** Proportion of *B. rapa* genome in progenies of the three categories of *B. juncea* parents. The orange, blue, and green boxplots represent genotypes A, B, and H, respectively. **d** Actual A^r^ genotype frequency for 132 SNP markers in 107 *B. juncea* introgression lines. The red line denotes the theoretical A^r^ genotype frequency.

**Figure 5:**
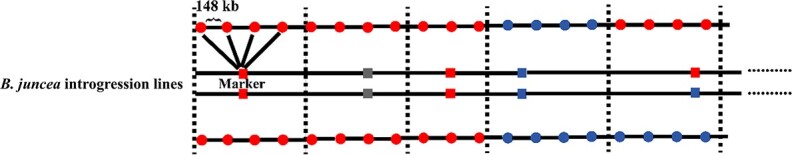
Schematic diagram of determination of genome-wide SNPs genotype based on marker genotyping results. Red, gray, and blue squares represent genotyping results of the markers, which were A^r^, missing, and A^j^. Circles represent the SNPs of the parents used for substitution.

**Figure 6:**
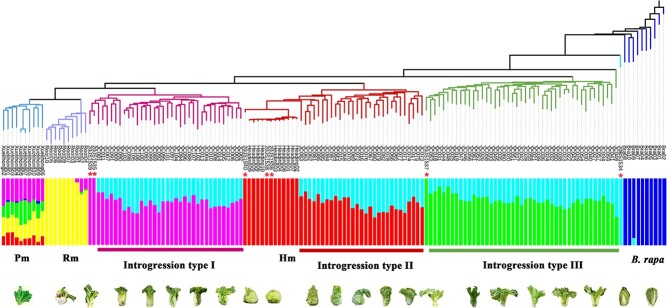
Phylogenetic tree and population structure (*K* = 6) of 154 individuals based on 1642 SNPs. Pm, Rm, and Hm represent potherb mustard, root mustard, and heading mustard accessions, respectively. Red asterisks represent parental materials. Introgression types I, II, and III represent the introgression progenies of semi-heading I, heading, and semi-heading II *B. juncea*.

**Figure 7:**
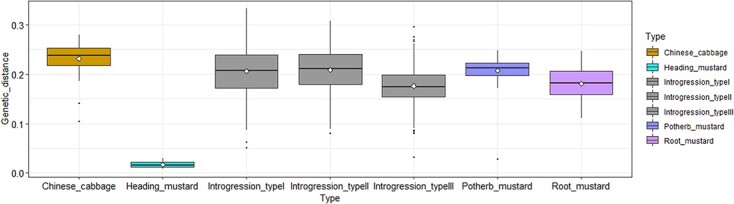
Genetic distance between each of the seven material clusters. The *B. juncea* introgression lines were divided into three types according to the phylogenetic tree (I, II, and III) and are represented by the gray box plots. The white diamonds represent the average genetic distance.

**Figure 8:**
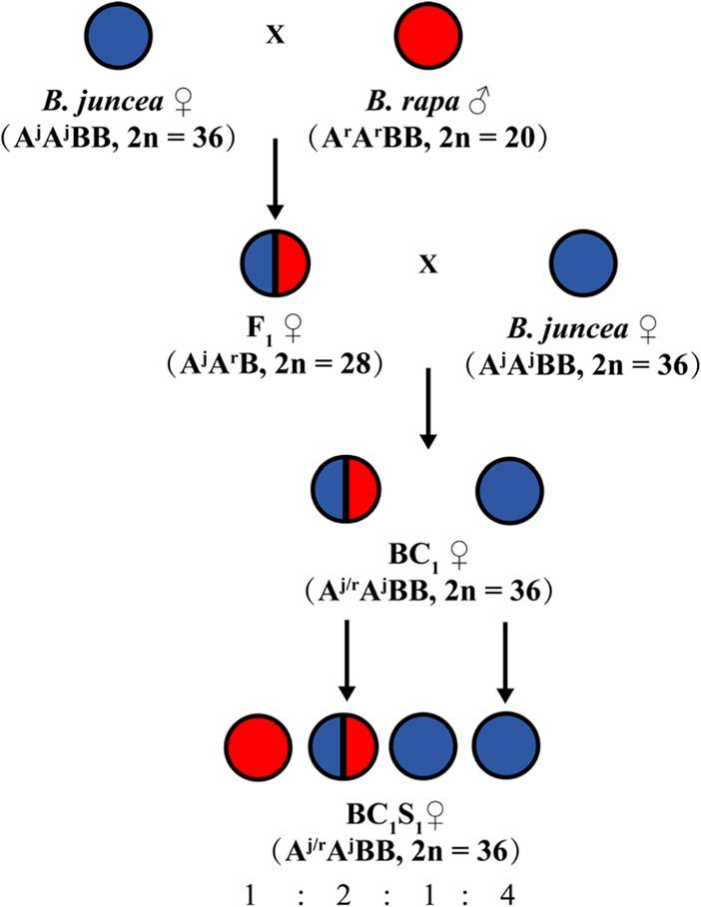
Strategy used for the development of *B. juncea* introgression lines.

### Detection of B genome in *Brassica juncea* introgression lines

To detect B chromosomes in the introgression lines, a strategy of screening B-genome markers was adopted. SNPs were detected by mapping clean data of *B. rapa* parental line B9008 and the six *B. juncea* parental lines (http://39.100.233.196:82/download_genome/datasets/pub/Bju_6_reseq/) to the *B. juncea* reference genome (http://brassicadb.cn/). We chose SNPs polymorphic between A and B homoeologous sites and transferable to KASP markers. One SNP per B-genome chromosome was used for designing KASP markers (KASP primer sequences are shown in Supplementary Table S1). The polymorphisms of the eight KASP markers were firstly verified in a set of randomly selected germplasm accessions containing 16 *B. rapa* and 16 *B. juncea* accessions together with one *B. nigra* accession (Supplementary Fig. S1). These eight KASP markers were then used to genotype all the *B. juncea* introgression lines, showing that all of them could amplify the fragments from both the A genome and the B genome. This result indicated that the eight B chromosomes very likely existed in the introgression lines (Fig. 2, Supplementary Fig. S2).

### Development of a set of SNP markers evenly distributed on 10 chromosomes of a genome

To distinguish the A genome of *B. juncea* (BjuA) from the A genome of *B. rapa* (BraA), SNP markers were developed. The clean reads of the parental lines were aligned to the *B. rapa* reference genome. A total of 186 484 SNP loci were obtained, of which 17 368 specific SNPs met the selection criteria of being homozygous and polymorphic between BjuA and BraA. For evaluation of the introgression lines, 203 SNPs were selected from 3121 loci with no variation in the flanking region; they were evenly distributed across 10 chromosomes of the A genome.

A total of 132 SNP markers showed expected genotypes between B9008 and the *B. juncea* parents. These markers also met the requirement that they could not be amplified in the *B. nigra* genome (KASP primer sequences are shown in Supplementary Table S2). The number of specific SNP markers on each chromosome ranged from 10 on A04, A05, and A08 to 17 on A03. The genome-wide average density of specific SNPs was 2.142 Mb, with a range of 1.481 Mb on A10 to 2.958 Mb on A05. Since physical distance could not fully reflect the genetic recombination rate, these SNP markers were further anchored to the genetic linkage map of *B. rapa*. These SNP markers were first determined based on their corresponding binmarkers on the linkage map, whereupon the genetic position of the binmarker was used to represent the genetic position of SNPs (Fig. 3). Using this method, we evaluated the genetic distribution of the 132 SNP markers. The genome-wide average genetic interval was 6.72 cM, with a range of 4.11 cM on A01 to 10.66 cM on A05 (Table 2). In general, the distribution of 132 SNP markers among the chromosomes was reasonable, although there were several large gaps (>20 cM), including gaps located on both ends of A01, one on A9, and one on A10.

### *Brassica rapa* genome segments tended be retained in introgression lines

In total, a set of 132 polymorphic SNP markers was genotyped to evaluate the introgression of *B. rapa* in 107 *B. juncea* introgression lines (Fig. 4a). The genotyping results were recorded as the following four types: genotypes consistent with *B. rapa* and *B. juncea* [A (A^r^A^r^, orange in Fig. 4) and B (A^j^A^j^, blue), respectively], heterozygous genotype [H (A^r^A^j^, green)], and the missing genotype (gray).

A total of 14 124 loci were genotyped, among which 1472 were A^r^A^r^, 5440 were A^j^A^j^, 6890 were A^r^A^j^, and 322 were missing. The proportions of genotypes A, B, and H were 10.67, 39.41, and 49.92%, respectively. The segregation ratio of 107 *B. juncea* introgression lines in the BC_1_S_1_ population showed extremely significant differences (*P* < .005) from the expected results (A:B:H = 1:5:2), indicating that the progenies tended to retain the *B. rapa* genome in the process of distant hybridization of *B. juncea* and *B. rapa*, and existed in the form of heterozygosity.

A violin plot was constructed to show the proportions of three genotypes in 107 *B. juncea* introgression lines (Fig. 4b). The proportion range of genotypes A, B, and H were 0 to 29.55%, 6.06 to 70.45%, and 13.64 to 89.39%, respectively. The loci showing the A^r^ genotype (A^r^A^r^ + A^r^A^j^) were identified as the segments replaced by the *B. rapa* genome. To determine whether the rate of introgression of BraA was affected by *B. juncea* parental lines, the proportions of A^r^ genotype (A^r^A^r^ + A^r^A^j^) in progenies from three *B. juncea* categories were compared. The average proportions of A genotype corresponding to the progenies obtained from heading, semi-heading I, and semi-heading II were 57.31, 59.57, and 60.34%, respectively. There was no obvious difference in the *B. rapa* segment retaining rate between heading and semi-heading *B. juncea* parental lines. On average, 59.2% of the A genome segments in the *B. juncea* introgression lines were covered by the *B. rapa* genome.

The theoretical A^r^ genotype frequency was 25% in the BC_1_S_1_ population. The actual A^r^ genotype frequency was analyzed by the following calculation formula: A^r^ genotype frequency = A × 2 + H/(A + B + H) × 2. The A^r^ genotype frequency of 129 out of 132 markers was greater than the theoretical value, indicating that 97.73% of the markers were positively selected. In contrast, the actual A^r^ genotype frequencies of the three remaining markers were 9% for A01_364, 1% for A04_22, and 11% for A08_215 (Fig. 4d). As shown by graphical genotyping of the progenies, a large number of *B. juncea* segments (A^j^A^j^) appeared in these three positions (Fig. 4a), suggesting that these *B. juncea* genome regions were not readily replaced by *B. rapa* segments.

### Genetic diversity in *Brassica juncea* introgression lines compared with traditional accessions

To evaluate the genetic diversity of the *B. juncea* introgression lines, phylogenetic analysis was performed at the genome-wide level using a collection of representative *B. juncea* and *B. rapa* accessions. Each of the 10 or ten accessions were selected from each of heading mustard, potherb mustard (*B. juncea* var. *multiceps*), root mustard (*B. juncea* var. *megarrhiza*), and Chinese cabbage. These 40 accessions were resequenced for SNP calling.

Genome-wide SNPs were obtained from the alignment of clean reads of all parents and 40 accessions with the *B. rapa* reference genome. We divided the reference genome into 2000 regions with an interval of ~148 kb. Overall, 1642 common SNPs from chromosomes A01 to A10 with homozygous genotype were identified in all parents and 40 accessions. The missing genotype of each *B. juncea* introgression line was filled according to its adjacent locus genotype, and the genotyping data of 132 markers of 107 *B. juncea* introgression lines was then replaced by 1642 SNPs of their parents (Fig. 5).

A dendrogram of 1642 SNPs of 154 investigated lines showed that all *B. juncea* introgression lines could be divided into three major types (introgression types I, II, and III), and each type was associated with its *B. juncea* parents. There was almost no diversification between heading mustard accessions (Hm), and the genetic diversity was much narrower than that of potherb mustard (Pm) and root mustard (Rm) accessions. The phylogenetic tree revealed that the *B. juncea* introgression lines were separated from the heading *B. juncea* accessions and exhibited rich genetic diversity (Fig. 6).

The population structure of these investigated materials was analyzed, and the minimum value of the coefficient of variation (CV) was obtained at *K* = 6 (Supplementary Fig. S3). At *K* = 6, the *B. juncea* introgression lines were classified into three groups, consistent with the result of the phylogenetic tree (Fig. 6).

For further comparison of the heading mustard accessions and the *B. juncea* introgression lines, we calculated the genetic distance. The maximum genetic distance detected in heading mustard accessions (Hm) was only 0.03. However, the maximum genetic distances of the progenies of semi-heading I *B. juncea* (introgression_type I), heading *B. juncea* (introgression_type II), and semi-heading II *B. juncea* (introgression_type III) were 0.33, 0.31, and 0.30, respectively. Compared with heading mustard accessions, the maximum genetic distance of the introgression lines was significantly increased (Fig. 7, Supplementary Tables S3 and S4).

## Discussion

We report a breeding approach for *B. juncea* through the introgression of one of its diploid contributor species, *B. rapa*. The genotyping of *B. juncea* introgression lines indicated large-scale segmental introgressions involving *B. rapa* genome segments substituting for the A genome of *B. juncea*. The *B. juncea* introgression lines exhibited expanded morphological variation and rich genetic diversity, proving that *B. rapa* has great potential for increasing the genetic diversity of *B. juncea*. The new *B. juncea* germplasm with known *B. rapa* genetic composition is a valuable resource that could be used to improve *B. juncea* varieties.

We developed a set of 132 specific SNP markers that could distinguish *B. rapa* and *B. juncea* parents, and these were evenly distributed across all 10 chromosomes. Some chromosomes, such as A01, which has gaps at both ends, would cause a deviation in the assessment of the introgression rate, but in general the evaluation by SNP markers could reflect the segmental introgression ratio in these introgression lines. Our work demonstrated that the analysis of BC_1_S_1_ segregating families with specific SNP markers was an effective and high-throughput solution to trace the introgression segments. Different proportions of *B. rapa* segments were detected regardless of categories of recipient parents. It has been suggested that the A genome of *B. rapa* and *B. juncea* showed visible differentiation [31]. Therefore, this set of polymorphic SNP markers might be universal and beneficial for future relevant research.

We observed strong selection in the genomic region for the *B. juncea* parent genotypes. Three markers (A01_364, A04_22, A08_215) were significantly different from the other markers, and their actual genotype frequencies were much lower than the theoretical value of 25%. This may be caused by telomeres on chromosomes. During the generation of the BC_1_S_1_, we collected seeds from plants that produced more seeds without artificial pollination. This is obviously strong selection for self-compatibility. We speculated that the skewed ratios of these markers might be associated with self-compatibility selection. The reported Al S-locus on chromosome 4 of *Arabidopsis thaliana* has three collinear fragments in *B. rapa*, which were located at A01, A03, and A08 in subgenomes LF, MF1, and MF2 [32]. However, variation in these markers was not seen among *B. juncea* and *B. rapa* parents, indicating the possibility of other unknown SI (self-incompatibility) genes, but we cannot exclude other factors that may be involved.

We used three different heading types of *B. juncea* accessions as recipient parents, and we expected the *B. juncea* introgression lines to be classified into three categories related to the recipients. Grouping based on the KASP markers allowed distinct separation of the *B. rapa* and *B. juncea* accessions, while the *B. juncea* introgression lines were in between these two parental species. The *B. juncea* introgression lines from three types of parents intersected each other and showed no group corresponding to their recipient parents (Supplementary Fig. S4). These results indicated that our rule to ensure the universality of the markers in distinguishing *B. rapa* from *B. juncea* decreased their power in resolving the variation within the two species. To properly assess the diversity of the derived progenies, we used a set of 1642 genome-wide SNPs. Their genotypes in the introgression lines were obtained based on the sources of the fragments determined by the KASP markers and the parental genotypes of the corresponding fragments. The phylogenetic tree and population genetic analysis of 1642 SNPs showed an association with the progenies of the three categories of *B. juncea* parents. The results also demonstrated increased diversity among the *B. juncea* introgression lines relative to normal *B. juncea* accessions. To further confirm this result, 188 *B. rapa* and 110 *B. juncea* accessions, together with the 10 accessions of each group used in the present study, were evaluated for their genetic diversity using the SNPs called from resequencing data (Chang et al.). The result was consistent with what we observed in the population composed of 10 of each group (Supplementary Fig. S5). Our strategy for evaluating genome-wide diversity avoided the need to sequence all used lines by referring to the genotype of flanking KASP markers. This strategy considerably decreased the expense of whole-genome diversity evaluation.

Several studies have noted the importance of creating more variability and broadening the genetic base of *B. juncea* by making crosses between genetically diverse parents [33–35]. However, to our knowledge, no other report has adopted the strategy of introducing genetic variations from their diploid contributor species. The present study created a set of novel *B. juncea* germplasms and systematically evaluated the expanded diversity at the whole-genome level. Using a set of genome-wide SNPs, we demonstrated that the genetic distance of the heading *B. juncea* was enhanced greatly from 0.03 to an estimated maximum of 0.33. Corresponding to increased genetic diversity, we also observed obviously increased phenotypical variations among the introgression lines. This introgression strategy can be extended to the improvement of other *Brassica* allotetraploid species.

## Materials and methods

### Plant materials

*Brassica rapa* B9008 is a line that has been assembled *de novo* and exhibits desirable characteristics such as self-compatibility, high seed-setting rate, early flowering, and a compact leafy head [36]. The BC_1_S_1_ population of the *B. juncea* introgression lines was derived from crosses between *B. rapa* B9008 and six accessions of heading *B. juncea* (three accessions of *B. juncea* were semi-heading; Table 3). All parent materials were provided by the Molecular Genetics Group, Institute of Vegetables and Flowers (IVF), and the Chinese Academy of Agricultural Sciences (CAAS).

*Brassica juncea* was used as the female parent, and B9008 was used as the male parent to produce the *F*_1_ generation through embryo culture [37]. The *F*_1_ hybrid plants were backcrossed with the recipient parents to generate the BC_1_ population. The plants in the backcrossed population were further self-pollinated and harvested to generate *B. juncea* introgression lines (Fig. 8). In August 2020, 10 seedlings per line were planted in the experimental field in Beijing. The plants were selected for the experiments according to agronomic traits such as the leafy head and resistance.

### Morphological observation

The leafy head of the *B. juncea* introgression lines and parent materials was photographed 3 months after transplanting. The outer leaves of the plants were removed for observation of the heading characteristics.

### DNA extraction and sequencing

Total genomic DNA was isolated from young leaves following the modified CTAB method [38]. The DNA samples for genotyping were diluted to a final concentration of 50 ng/μl. DNA from seven parental lines was sequenced using a BGISEQ-500 sequencer and the Illumina NovaSeq 6000 platform with paired ends. We used fastp [39] to filter low-quality reads, reads with >10% undetected bases, and reads with adapters to obtain high-quality reads (clean data).

### SNP calling for KASP marker design

SNP calling was conducted by aligning the clean data of each parent material to the *B. rapa* reference genome ‘Chiifu’, the *B. nigra* reference genome, and the *B. juncea* reference genome (www.brassicadb.cn) with the GATK software toolkit [40, 41].

The A genome of *B. rapa* and that of *B. juncea* were named BraA and BjuA, respectively, and the B genome of *B. juncea* was named BjuB. The acquisition of polymorphic SNPs between the A genome (BraA and BjuA) and BjuB was based on the SNP set uniform in BraA and BjuA of the *B. juncea* reference genome, but polymorphic between BraA and BjuB of the *B. juncea* reference genome, while the *B. juncea* parental lines were not polymorphic in either the A or the B genome of the *B. juncea* reference genome.

The selected ‘BraA/BjuA’ allele-specific SNPs from custom-written Python scripts had the following criteria: (i) homozygous for both parents; (ii) no polymorphism between BraA and the *B. rapa* reference genome; (iii) polymorphic between BjuA and BraA; (iv) uniform in *B. juncea* parental lines; (v) no sequence variation within the 100-bp flanking region; (vi) no homologous region in the *B. nigra* genome; and (vii) relatively even distribution on chromosomes.

The flanking sequence of each selected SNP was further used to search for the unique hit to the reference genomes of *B. rapa* and *B. juncea*. Forward primers (A1/A2) and common reverse primers (C) were designed according to the manufacturer’s instructions (LGC Genomics) using Primer3 software.

### SNP genotyping through KASP

The KASP genotyping assay was used to detect specific SNPs. KASP amplifications were performed in a 384-well format using the Scientific QuantStudio™ 12K Flex Real-Time PCR System (Thermo Fisher Scientific, USA). A final reaction volume of 5 μl was used and contained 2.5 μl 2 × KASP V4.0 Master Mix (LGC Genomics), 2.5 μl DNA template, and 0.07 μl KASP assay mix. Non-template controls were included in each SNP reaction plate. The PCR program was performed as described by LGC. The temperature of the fluorescence measurement was 30°C and the time was 1 min. The genotype clusters were viewed with SNPviewer software (LGC Genomics, https://www.biosearchtech.com/support/tools/genotyping-software/snpviewer).

### Genetic analysis

For genetic map construction, the raw data of Chinese cabbage DH lines were aligned to the *B. rapa* reference genome for calling SNPs by BWA and SAMtools [42–45]. Recombination bins were constructed as genetic markers and imported into JoinMap 4.0 software, and the physical distance of the binmarkers against the genetic distance was obtained. The genetic map and graphical genotyping analysis were performed using R software. To observe the phylogenetic relationship of the *B. juncea* introgression lines, the phylogenetic tree was constructed by applying the neighbor-joining method and the Interactive Tree of Life (iTOL) [46]. The population structure was further inferred with ADMIXTURE. The number of subpopulations analyzed ranged from *K* = 1 to *K* = 7 [47].

## Acknowledgements

This work was supported by the China Agriculture Research System of Ministry of Finance (MOF). and Ministry of Agriculture and Rural Affairs (MARA), and the Agricultural Science and Technology Innovation Program (ASTIP).

## Author contributions

X.W. and J.W. designed the project. X.L. and L.Z. generated the materials and conducted the experiments. L.C. generated the sequencing data. L.Z., L.C., and T.W. performed the data analysis. L.Z., J.W., and X.W. wrote the manuscript.

## Data availability

The parental lines are available upon request. The clean data of six *B. juncea* parental lines as well as the sequence polymorphism data of 40 representative *B. juncea* and *B. rapa* accessions are available and have been deposited in BRAD (www.brassicadb.cn).

## Conflict of interest

The authors declare that they have no conflict of interest.

## Supplementary data

Supplementary data is available at *Horticulture Research* online.

## Supplementary Material

Web_Material_uhab054

## References

[1] Nagaharu U . Genome analysis in *Brassica* with special reference to the experimental formation of *B. napus* and peculiar mode of fertilization. Jpn J Bot. 1935;7:389–452.

[2] Chen S , Wan Z, Nelson MN et al. Evidence from genome-wide simple sequence repeat markers for a polyphyletic origin and secondary centers of genetic diversity of *Brassica juncea* in China and India. J Hered. 2013;104:416–27. 23519868 10.1093/jhered/est015

[3] YAO QL , Chen FB, Fang P et al. Genetic diversity of Chinese vegetable mustard (*Brassica juncea* Coss) landraces based on SSR data. Biochem Syst Ecol. 2012;45:41–8.

[4] Chen FB , Liu HF, Yao QL et al. Evolution of mustard (*Brassica juncea* Coss) subspecies in China: evidence from the chalcone synthase gene. Genet Mol Res. 2016;15:2. 10.4238/gmr.15028045 27173323

[5] Gao LW , Lyu SW, Tang J et al. Genome-wide analysis of auxin transport genes identifies the hormone responsive patterns associated with leafy head formation in Chinese cabbage. Sci Rep. 2017;7:42229. 28169368 10.1038/srep42229 PMC5294403

[6] Yu J , Gao L, Liu W et al. Transcription coactivator ANGUSTIFOLIA3 (AN3) regulates leafy head formation in Chinese cabbage. Front Plant Sci. 2019;10:520. 31114598 10.3389/fpls.2019.00520 PMC6502973

[7] Cheng F , Sun R, Hou X et al. Subgenome parallel selection is associated with morphotype diversification and convergent crop domestication in *Brassica rapa* and *Brassica oleracea*. Nat Genet. 2016;48:1218–24. 27526322 10.1038/ng.3634

[8] Payal B , Shashi B, Banga SS et al. Heterosis as investigated in terms of polyploidy and genetic diversity using designed *Brassica juncea* amphiploid and its progenitor diploid species. PLoS One. 2012;7:2. 10.1371/journal.pone.0029607 PMC3283606 22363404

[9] Yadava SK , Arumugam N, Mukhopadhyay A et al. QTL mapping of yield-associated traits in *Brassica juncea*: meta-analysis and epistatic interactions using two different crosses between east European and Indian gene pool lines. Theor Appl Genet. 2012;125:1553–64. 22821338 10.1007/s00122-012-1934-3

[10] Li Q , Mei J, Zhang Y et al. A large-scale introgression of genomic components of *Brassica rapa* into *B. napus* by the bridge of hexaploid derived from hybridization between *B. napus* and *B. oleracea*. Theor Appl Genet. 2013;126:22073–80. 10.1007/s00122-013-2119-4 23699961

[11] Hasan MJ , Rahman H. Resynthesis of *Brassica juncea* for resistance to *Plasmodiophora brassicae* pathotype 3. Breed Sci. 2018;68:385–91. 30100807 10.1270/jsbbs.18010 PMC6081302

[12] Chen ZJ . Genetic and epigenetic mechanisms for gene expression and phenotypic variation in plant polyploids. Annu Rev Plant Biol. 2007;58:377–406. 17280525 10.1146/annurev.arplant.58.032806.103835 PMC1949485

[13] Chauhan JS , Singh KH, Singh VV et al. Hundred years of rapeseed-mustard breeding in India: accomplishments and future strategies. Indian J Agric Sci. 2011;81:1093–109.

[14] Kumar S , Hilario E, Deng CH et al. Turbocharging introgression breeding of perennial fruit crops: a case study on apple. Hortic Res. 2020;7:1–7. 32257233 10.1038/s41438-020-0270-z PMC7109137

[15] Bennett RA , Seguin-Swartz G, Rahman H. Broadening genetic diversity in canola using the C-genome species *Brassica oleracea* L. Crop Sci. 2012;52:2030–2039.

[16] Chen S , Nelson MN, Chèvre AM et al. Trigenomic bridges for *Brassica* improvement. Crit Rev Plant Sci. 2011;30:524–47.

[17] Mason AS , Batley J. Creating new interspecific hybrid and polyploid crops. Trends Biotechnol. 2015;33:436–41. 26164645 10.1016/j.tibtech.2015.06.004

[18] Karim MM , Siddika A, Tonu N et al. Production of high yield short duration *Brassica napus* by interspecific hybridization between *B. oleracea* and *B. rapa*. Breed Sci. 2014;63:495–502. 24757390 10.1270/jsbbs.63.495 PMC3949587

[19] Chatterjee D , Banga S, Gupta M et al. Resynthesis of *Brassica napus* through hybridization between *B. juncea* and *B. carinata*. Theor Appl Genet. 2016;129:977–90. 26849238 10.1007/s00122-016-2677-3

[20] Narasimhulu SB , Kirti PB, Prakash S et al. Resynthesis of *Brassica carinata* by protoplast fusion and recovery of a novel cytoplasmic hybrid. Plant Cell Rep. 1992;11:428–32. 24201548 10.1007/BF00234376

[21] Mei J , Shao C, Yang R et al. Introgression and pyramiding of genetic loci from wild *Brassica oleracea* into *B. napus* for improving *Sclerotinia* resistance of rapeseed. Theor Appl Genet. 2020;133:1313–9. 32008057 10.1007/s00122-020-03552-w

[22] Guo N , Cheng F, Wu J et al. Anthocyanin biosynthetic genes in *Brassica rapa*. BMC Genomics. 2011;15:135–42. 10.1186/1471-2164-15-426 PMC4072887 24893600

[23] Su TB , Wang W, Li P et al. Genomic variation map provides insights into the genetic basis of spring Chinese cabbage (*Brassica rapa* ssp. *pekinensis*). Plant Commun. 2018;11:1360–76. 10.1016/j.molp.2018.08.006 30217779

[24] Ramesh P , Mallikarjuna G, Sameena S et al. Advancements in molecular marker technologies and their applications in diversity studies. J Biosci. 2020;45:123. 33097680

[25] Navabi ZK , Stead KE, Pires JC et al. Analysis of B-genome chromosome introgression in interspecific hybrids of *Brassica napus* × *B. carinata*. Genetics. 2011;187:659–73. 21196520 10.1534/genetics.110.124925 PMC3063663

[26] Badaeva ED , Amosova AV, Goncharov NP et al. A set of cytogenetic markers allows the precise identification of all A-genome chromosomes in diploid and polyploid wheat. Cytogenet Genome Res. 2015;146:71–9. 26160023 10.1159/000433458

[27] Megyeri M , Mikó P, Farkas A et al. Cytomolecular discrimination of the A^m^ chromosomes of *Triticum monococcum* and the A chromosomes of *Triticum aestivum* using microsatellite DNA repeats. J Appl Genet. 2017;58:67–70. 27468932 10.1007/s13353-016-0361-6

[28] Li F , Kitashiba H, Inaba K et al. A *Brassica rapa* linkage map of EST-based SNP markers for identification of candidate genes controlling flowering time and leaf morphological traits. DNA Res. 2009;16:311–23. 19884167 10.1093/dnares/dsp020 PMC2780953

[29] Wang X , Yu K, Li H et al. High-density SNP map construction and QTL identification for the apetalous character in *Brassica napus* L. Front Plant Sci. 2015;6:1164. 26779193 10.3389/fpls.2015.01164 PMC4688392

[30] Steele KA , Quinton-Tulloch MJ, Amgai RB et al. Accelerating public sector rice breeding with high-density KASP markers derived from whole genome sequencing of *indica* rice. Mol Breed. 2018;38:1–13. 10.1007/s11032-018-0777-2 PMC5842261 29563850

[31] Yang JH , Liu D, Wang X et al. The genome sequence of allopolyploid *Brassica juncea* and analysis of differential homoeolog gene expression influencing selection. Nat Genet. 2016;48:1225–32. 27595476 10.1038/ng.3657

[32] Cui YN , Zhuang M, Wu J et al. Segmental translocation contributed to the origin of the *Brassica* S-locus. Hortic Plant J. 2020;6:167–78.

[33] Gupta CM , Banga SS. Cytogenetic stability and genome size variations in newly developed derived *Brassica juncea* allopolyploid lines. J Oilseed Brassica. 2016;5:118–27.

[34] Zhang K , Wang XW, Cheng F. Plant polyploidy: origin, evolution, and its influence on crop domestication. Hortic Plant J. 2019;5:231–9.

[35] Sharma D , Nanjundan J, Singh L et al. Genetic diversity in leafy mustard (*Brassica juncea* var. *rugosa*) as revealed by agro-morphological traits and SSR markers. Physiol Mol Biol Plants. 2020;26:2005–18. 33088045 10.1007/s12298-020-00883-2 PMC7548306

[36] Cai X , Chang L, Zhang T et al. Impacts of allopolyploidization and structural variation on intraspecific diversification in *Brassica rapa*. Genome Biol. 2021;22:166. 34059118 10.1186/s13059-021-02383-2 PMC8166115

[37] Wen J , Tu J, Fu TD et al. Improving ovary and embryo culture techniques for efficient resynthesis of *Brassica napus* from reciprocal crosses between yellow-seeded diploids *B. rapa* and *B. oleracea*. Euphytica. 2008;162:81–9.

[38] Porebski S , Bailey LG, Baum BR. Modification of CTAB DNA extraction protocol for plants containing high polysaccharide and polyphenol components. Plant Mol Biol Report. 1997;15:8–15.

[39] Chen SF , Zhou YQ, Chen Y et al. Fastp: an ultra-fast all-in-one FASTQ preprocessor. Bioinformatics. 2018;34:i884–90. 30423086 10.1093/bioinformatics/bty560 PMC6129281

[40] Wang XW , Wang H, Wang J et al. The genome of the mesopolyploid crop species *Brassica rapa*. Nat Genet. 2011;43:1035–9. 21873998 10.1038/ng.919

[41] McKenna A , Hanna M, Banks E et al. The genome analysis toolkit: a MapReduce framework for analyzing next-generation DNA sequencing data. Genome Res. 2010;20:1297–303. 20644199 10.1101/gr.107524.110 PMC2928508

[42] Li H , Durbin R. Fast and accurate long-read alignment with Burrows–Wheeler transform. Bioinformatics. 2010;26:589–95. 20080505 10.1093/bioinformatics/btp698 PMC2828108

[43] Li H , Handsaker B, Wysoker A et al. The sequence alignment/map format and SAMtools. Bioinformatics. 2009;25:2078–9. 19505943 10.1093/bioinformatics/btp352 PMC2723002

[44] Liu J , Bo L, Feng C et al. A high density linkage map facilitates QTL mapping of flowering time in *Brassica rapa*. Hortic Plant J. 2016;2:217–23.

[45] Zhang L , Cai X, Wu J et al. Improved *Brassica rapa* reference genome by single-molecule sequencing and chromosome conformation capture technologies. Hortic Res. 2018;5:50. 30131865 10.1038/s41438-018-0071-9 PMC6092429

[46] Letunic I , Bork P. Interactive tree of life (iTOL) v4: recent updates and new developments. Nucleic Acids Res. 2019;47:W256–9. 30931475 10.1093/nar/gkz239 PMC6602468

[47] Alexander DH , Novembre J, Lange K. Fast model-based estimation of ancestry in unrelated individuals. Genome Res. 2009;19:1655–64. 19648217 10.1101/gr.094052.109 PMC2752134

