# Supplementary material for: Expanding the genetic variation of Brassica juncea by introgression of the Brassica rapa genome
Source: Hortic Res. 2022 Jan 19;9:uhab054. doi: 10.1093/hr/uhab054 (PMC8883073; doi:10.1093/hr/uhab054)
Supplement: Web_Material_uhab054 [file web_material_uhab054.docx]

**Tables**

Supplementary Table 1 KASP primer sequences for detection of B-genome

| Marker name | Locations on the B-genome of B. juncea | Locations on the A-genome of B. juncea | Sequence(A1/A2/C) |
| --- | --- | --- | --- |
| B01_40 | B01_4006868 | A04_10539597 | GAAGGTGACCAAGTTCATGCTAACATTAGTTTATGACCTTGAGGAGGG  GAAGGTCGGAGTCAACGGATTAACATTAGTTTATGACCTTGAGGAGGC  CCTCGCTTCTCTGCAGGAAG |
| B02_248 | B01_24883920 | A10_10106328 | GAAGGTGACCAAGTTCATGCTAGACAAGTATCTAGCCTGCTAAGA  GAAGGTCGGAGTCAACGGATTAGACAAGTATCTAGCCTGCTAAGG  CTTGAAACCTCTCTGATTCCATGG |
| B03_38 | B01_3863100 | A08_16458778 | GAAGGTGACCAAGTTCATGCTCCACACTAACGCCACATTTGC  GAAGGTCGGAGTCAACGGATTCCACACTAACGCCACATTTGG  TTTCCGGATTCAACGATGACAATG |
| B04_11 | B01_11155192 | A02_33661364 | GAAGGTGACCAAGTTCATGCTCTTCCATGCAGCTCAAGGATCCT  GAAGGTCGGAGTCAACGGATTCTTCCATGCAGCTCAAGGATCCC  TATAACAGTACCCCTCATCCGAGA |
| B05_62 | B01_6241110 | A01_4733655 | GAAGGTGACCAAGTTCATGCTGCAAACTCCGAGCCTTTGCA  GAAGGTCGGAGTCAACGGATTGCAAACTCCGAGCCTTTGCC TGAGATTGAAACACCAGCAAGAAC |
| B06_81 | B01_8128585 | A06_18881497 | GAAGGTGACCAAGTTCATGCTCTCCAAGCCACTCTCCTTAGGTAG  GAAGGTCGGAGTCAACGGATTCTCCAAGCCACTCTCCTTAGGTAT  GCTACTGCTGTCGAATTCTCAAAA |
| B07_3 | B01_323688 | A01_42643245 | GAAGGTGACCAAGTTCATGCTTGCAATTTGAAGATTTCTTTGTCCTG  GAAGGTCGGAGTCAACGGATTTGCAATTTGAAGATTTCTTTGTCCTC  TGTCTTATGTCAATTCGTCCAAGC |
| B08_126 | B01_126686562 | A03_5801058 | GAAGGTGACCAAGTTCATGCTCAGAGACATAAGAAAGGAAATACCAGAAT  GAAGGTCGGAGTCAACGGATTCAGAGACATAAGAAAGGAAATACCAGAAC  CCGAGTCTCTTGGTTCAGACATTA |

Supplementary Table 2 KASP primer sequences for distinguishing BjuA from BraA

| Marker name | Locations on the *B. rapa* chromosome | Locations on the *B. juncea* chromosome | Sequence(A1/A2/C) |
| --- | --- | --- | --- |
| A01_28 | A01_2961237 | A01_2879092 | GAAGGTGACCAAGTTCATGCTTCAGAAGTGGTCCATTAGATGATC  GAAGGTCGGAGTCAACGGATTTCAGAAGTGGTCCATTAGATGATA  AAATCAGGACCACAAAACAACACA |
| A01_40 | A01_4685000 | A01_4016706 | GAAGGTGACCAAGTTCATGCTGAATGCAACGATAGCCATCACTG  GAAGGTCGGAGTCAACGGATTGAATGCAACGATAGCCATCACTA  TATTCACTATACTCAACACGCGGT |
| A01_63 | A01_5733337 | A01_6373302 | GAAGGTGACCAAGTTCATGCTAGATTACATGTCATATTCTCAGTGCC  GAAGGTCGGAGTCAACGGATTAGATTACATGTCATATTCTCAGTGCT  GCTTCTTCCGCTTTATACAAGGAC |
| A01_87 | A01_7801379 | A01_8790159 | GAAGGTGACCAAGTTCATGCTGGTAGAGTTTTGTTGTTTACACGTG  GAAGGTCGGAGTCAACGGATTGGTAGAGTTTTGTTGTTTACACGTA  ATTCAAGAGGATCCTAAGATGCGT |
| A01_105 | A01_9389394 | A01_10586993 | GAAGGTGACCAAGTTCATGCTACGAGGCATGATTTAAGGTTTTGTT  GAAGGTCGGAGTCAACGGATTACGAGGCATGATTTAAGGTTTTGTC  CCCATTTGTTGTTCAGCTTTGTTG |
| A01_115 | A01_ 10243750 | A01_11569804 | GAAGGTGACCAAGTTCATGCTTGTGATCTATGAATTTTTAGTTGTTCAACA  GAAGGTCGGAGTCAACGGATTTGTGATCTATGAATTTTTAGTTGTTCAACC  AGATGCATGTATTGATGGTTTAATGT |
| A01_150 | A01_ 16542525 | A01_15042165 | GAAGGTGACCAAGTTCATGCTTTTCCTGCTAGCCAAATACTGTTG  GAAGGTCGGAGTCAACGGATTTTTCCTGCTAGCCAAATACTGTTT  CGGTACAAATCCATATGTGACTGC |
| A01_176 | A01_ 15749510 | A01_17664154 | GAAGGTGACCAAGTTCATGCTTCCTCTGTAGGTTGGAAATATTGGT  GAAGGTCGGAGTCAACGGATTTCCTCTGTAGGTTGGAAATATTGGA  GTAACTTTTGAACATCAGGTGCCA |
| A01_213 | A01_ 18008949 | A01_21398848 | GAAGGTGACCAAGTTCATGCTTCCTATCTGCGCGTTGGC |
|  |  |  | GAAGGTCGGAGTCAACGGATTTCCTATCTGCGCGTTGGT  GATATGACCACCAACATCACCGAA |
| A01_233 | A01_ 18655565 | A01_23317709 | GAAGGTGACCAAGTTCATGCTTAGTGATGGTCATAGCTCAAATCC |
|  |  |  | GAAGGTCGGAGTCAACGGATTTAGTGATGGTCATAGCTCAAATCA |
|  |  |  | AATGTTCGATGATAGTTTCCAGCA |
| A01_252 | A01_ 20208678 | A01_25234255 | GAAGGTGACCAAGTTCATGCTTCTAAACAGCTAATCATTCAGAACTAAC  GAAGGTCGGAGTCAACGGATTTCTAAACAGCTAATCATTCAGAACTAAT |
|  |  |  | AACGTCATAAAAGATTGCGGTCAG |
| A01_278 | A01_ 22734972 | A01_27898021 | GAAGGTGACCAAGTTCATGCTAAGTGAAACGTGTTCTTCTCCTCG  GAAGGTCGGAGTCAACGGATTAAGTGAAACGTGTTCTTCTCCTCA  AACCTTACTGGGAGATGCTTCTTT |
| A01_303 | A01_ 22473115 | A01_30350550 | GAAGGTGACCAAGTTCATGCTCTACGAGGCATGAAACCGAAGATG |
|  |  |  | GAAGGTCGGAGTCAACGGATTCTACGAGGCATGAAACCGAAGATA |
|  |  |  | AGACTTTGTTACAGCTGTGCAATC |
| A01_339 | A01_ 24334633 | A01_33998968 | GAAGGTGACCAAGTTCATGCTCGTGAAAGAGTGGCCTTAAGTTCA  GAAGGTCGGAGTCAACGGATTCGTGAAAGAGTGGCCTTAAGTTCG  CTGAATCTGAGCCGAAATGTGTTT |
| A01_364 | A01_ 26047475 | A01_36489590 | GAAGGTGACCAAGTTCATGCTGAGAGAGAGAGAGCGAACCTCAAT |
|  |  |  | GAAGGTCGGAGTCAACGGATTGAGAGAGAGAGAGCGAACCTCAAC |
|  |  |  | TTGCTGTATCTTCTTCCACTCTCC |
| A02_12 | A02_ 10992540 | A02_12174113 | GAAGGTGACCAAGTTCATGCTCATTTGCAATAAACAAACCGGCC |
|  |  |  | GAAGGTCGGAGTCAACGGATTCATTTGCAATAAACAAACCGGCA |
|  |  |  | ACACACTGAAATACTAAAAACGGT |
| A02_19 | A02_ 1783989 | A02_1901911 | GAAGGTGACCAAGTTCATGCTCGATCACCGAGCAACACTAGAG  GAAGGTCGGAGTCAACGGATTCGATCACCGAGCAACACTAGAT  ATCTACTACGACCATCACCGGTTA |
| A02_36 | A02_ 3258056 | A02_3608996 | GAAGGTGACCAAGTTCATGCTCGACCCTATGTTTTTACTTATATCACAAAG  GAAGGTCGGAGTCAACGGATTCGACCCTATGTTTTTACTTATATCACAAAA  CAGCTCTCCCATTAGCTTATGCTA |
| A02_52 | A02_ 4697406 | A02_5209708 | GAAGGTGACCAAGTTCATGCTTCAAATTCTGAGCAGTTTCATCAGTC  GAAGGTCGGAGTCAACGGATTTCAAATTCTGAGCAGTTTCATCAGTT  GCCACTTGTTGATTTGAGAAGACA |
| A02_72 | A02_ 6509453 | A02_7201180 | GAAGGTGACCAAGTTCATGCTTTCTCGTTTGGTTTGTTTCGTTAA  GAAGGTCGGAGTCAACGGATTTTCTCGTTTGGTTTGTTTCGTTAC  TACACAATTTGCTCGTCAGTTTCG |
| A02_97 | A02_ 8797638 | A02_9771258 | GAAGGTGACCAAGTTCATGCTGGTGTTTATGACTTCTTGGTAACCATC  GAAGGTCGGAGTCAACGGATTGGTGTTTATGACTTCTTGGTAACCATT  TCTCATCGTCATATCCAGTGTTCC |
| A02_131 | A02_ 11891293 | A02_13132342 | GAAGGTGACCAAGTTCATGCTGGTTACCATAAAAATGTCTGACTAAAACA  GAAGGTCGGAGTCAACGGATTGGTTACCATAAAAATGTCTGACTAAAACT  CGTTTTTGAAAACCAACATCTCGT |
| A02_147 | A02_ 13414493 | A02_14789185 | GAAGGTGACCAAGTTCATGCTTAGGCATTACCAACAGTGTGATTA  GAAGGTCGGAGTCAACGGATTTAGGCATTACCAACAGTGTGATTG  ACATCGTGCTTTCAGGTCATTTTT |
| A02_261 | A02_ 24164132 | A02_26172729 | GAAGGTGACCAAGTTCATGCTTGGGAAGCAGCCAGAGGTA  GAAGGTCGGAGTCAACGGATTTGGGAAGCAGCCAGAGGTT  TAAGAGCAATCAGACACTTGGACA |
| A02_298 | A02_ 24398363 | A02_29876481 | GAAGGTGACCAAGTTCATGCTGCCATAGCCATAGCCAAAGCC  GAAGGTCGGAGTCAACGGATTGCCATAGCCATAGCCAAAGCT  AAGTCAACTTGTTCCTGAATTGCC |
| A02_330 | A02_ 28108898 | A02_33004767 | GAAGGTGACCAAGTTCATGCTACAAACGTGATGATTCTGGTTACAG  GAAGGTCGGAGTCAACGGATTACAAACGTGATGATTCTGGTTACAT  GAAGAAACAGAGCATGTGACACAA |
| A02_371 | A02_ 30858707 | A02_37109514 | GAAGGTGACCAAGTTCATGCTAGTTCACTATTGATTTGCTAAATGAGATAG  GAAGGTCGGAGTCAACGGATTAGTTCACTATTGATTTGCTAAATGAGATAC  AAATAATAGTGCGAGCCGAAATGG |
| A03_16 | A03_1452121 | A03_1627126 | GAAGGTGACCAAGTTCATGCTAACCCTTTGAAAAAGTTACTTATAAGTTT  GAAGGTCGGAGTCAACGGATTAACCCTTTGAAAAAGTTACTTATAAGTTG  TGCACGTACAATATCTATAGTCGA |
| A03_364 | A03_3385609 | A03_3649446 | GAAGGTGACCAAGTTCATGCTGCTCAGAAAATACAGACCTCTCACTAA  GAAGGTCGGAGTCAACGGATTGCTCAGAAAATACAGACCTCTCACTAG  TGATACAGGACACACTGACAAGAG |
| A03_61 | A03_4926783 | A03_6184157 | GAAGGTGACCAAGTTCATGCTGTCAAAGCATTCGGTTTTTCGGT  GAAGGTCGGAGTCAACGGATTGTCAAAGCATTCGGTTTTTCGGG  CCAGCTCAAGCAAGGTTTTCATAA |
| A03_82 | A03_6809208 | A03_8223265 | GAAGGTGACCAAGTTCATGCTAGCAAATCACTAATAACCTCAAGTCT  GAAGGTCGGAGTCAACGGATTAGCAAATCACTAATAACCTCAAGTCC  GGGTTACCAAATCCTTCTCTGAGT |
| A03_103 | A03_8568041 | A03_10304310 | GAAGGTGACCAAGTTCATGCTTGACTGATGTAAAGTTGGACAAAC  GAAGGTCGGAGTCAACGGATTTGACTGATGTAAAGTTGGACAAAT  TGAAAGAAGAAGGTGGAAAGGAGA |
| A03_119 | A03_ 9846302 | A03_11944783 | GAAGGTGACCAAGTTCATGCTTCACTTTGAATAATGCTGCAAGAAT  GAAGGTCGGAGTCAACGGATTTCACTTTGAATAATGCTGCAAGAAA  GCCTAGGCCCATTACCAAGTAATA |
| A03_132 | A03_ 11147605 | A03_13205765 | GAAGGTGACCAAGTTCATGCTTGAGATACCTACTGCTTGTTTCAG  GAAGGTCGGAGTCAACGGATTTGAGATACCTACTGCTTGTTTCAC  GGCTTTGCTTAGAGACCAATATGA |
| A03_157 | A03_ 13755121 | A03_15773204 | GAAGGTGACCAAGTTCATGCTATTAAAATCCAACCCCACAATATCAGAC  GAAGGTCGGAGTCAACGGATTATTAAAATCCAACCCCACAATATCAGAT  TGGGGGTCCATCAAGAGGC |
| A03_177 | A03_ 15033907 | A03_17740918 | GAAGGTGACCAAGTTCATGCTTCTCAAATGAAGCAAGCTATAGTGTG  GAAGGTCGGAGTCAACGGATTTCTCAAATGAAGCAAGCTATAGTGTA  CTCTCCACCCCCATTAAACTTTTG |
| A03_196 | A03_ 16750629 | A03_19674520 | GAAGGTGACCAAGTTCATGCTCTCTACATTGCTTCCCAATTCGC  GAAGGTCGGAGTCAACGGATTCTCTACATTGCTTCCCAATTCGA  GAGCTTGTTGAAAAATATGGGGCT |
| A03_227 | A03_ 19575288 | A03_22795153 | GAAGGTGACCAAGTTCATGCTAACAAATTATAATTAGTTCAATCACCAATTG  GAAGGTCGGAGTCAACGGATTAACAAATTATAATTAGTTCAATCACCAATTA  CGTAGAACGGGCTTACCTTAGTTA |
| A03_236 | A03_ 20344007 | A03_23681456 | GAAGGTGACCAAGTTCATGCTGAGGAGCCGGCTTTTTGAGATG  GAAGGTCGGAGTCAACGGATTGAGGAGCCGGCTTTTTGAGATT  GCGGAGAGGAATTGTAGATCTGAA |
| A03_264 | A03_ 21229318 | A03_26476308 | GAAGGTGACCAAGTTCATGCTTTTTTGTTCAGAGTGGGCATG  GAAGGTCGGAGTCAACGGATTTTTTTGTTCAGAGTGGGCATA  ATGGAATCAAATCGGGTATCTCGT |
| A03_283 | A03_ 22586105 | A03_28359682 | GAAGGTGACCAAGTTCATGCTAGAATGTTATCGTCCGAGTAATTCC  GAAGGTCGGAGTCAACGGATTAGAATGTTATCGTCCGAGTAATTCT  GTATTGACCGGAATTGAGTCCAAC |
| A03_305 | A03_ 24577429 | A03_30569183 | GAAGGTGACCAAGTTCATGCTCTCCTCTCTTCGTATTCTAACGGA  GAAGGTCGGAGTCAACGGATTCTCCTCTCTTCGTATTCTAACGGT  AAATTAAAAGCCGACGATGACGAA |
| A03_327 | A03_ 26497936 | A03_32751920 | GAAGGTGACCAAGTTCATGCTGAACCTTACCAGAGTCAGAATCCC  GAAGGTCGGAGTCAACGGATTGAACCTTACCAGAGTCAGAATCCG  TTCATCCATGATAACCGTTTTCGA |
| A03_353 | A03_ 28811990 | A03_35386755 | GAAGGTGACCAAGTTCATGCTTGATAATGTCAGTGCCGTCCAG  GAAGGTCGGAGTCAACGGATTTGATAATGTCAGTGCCGTCCAA  CAGGCATCGTTTACCCATATATGC |
| A04_22 | A04_ 1107554 | A04_2241960 | GAAGGTGACCAAGTTCATGCTGCTCCACCCTGCTTCTCTG  GAAGGTCGGAGTCAACGGATTGCTCCACCCTGCTTCTCTA  AAGAGGCTTTGTGTGGGAGAATAT |
| A04_34 | A04_ 3159791 | A04_3469080 | GAAGGTGACCAAGTTCATGCTGATTGTTGGGTTCTCGCATCACTC  GAAGGTCGGAGTCAACGGATTGATTGTTGGGTTCTCGCATCACTT  CGATCACTCTTCATCCTGTCAAGA |
| A04_52 | A04_ 4860072 | A04_5207919 | GAAGGTGACCAAGTTCATGCTTCCTTCAAGAAAACTTATCTTTGCG  GAAGGTCGGAGTCAACGGATTTCCTTCAAGAAAACTTATCTTTGCT  AGCTAGTATGCTCATTGTAACGGA |
| A04_65 | A04_6155578 | A04_6592845 | GAAGGTGACCAAGTTCATGCTCCACGAGAGGCATGAGGC  GAAGGTCGGAGTCAACGGATTCCACGAGAGGCATGAGGG  AAATGAACAGAAACGGAGAGAGTG |
| A04_102 | A04_10763092 | A04_10268057 | GAAGGTGACCAAGTTCATGCTTGCTTGAGCTGAGCAGTTTCA  GAAGGTCGGAGTCAACGGATTTGCTTGAGCTGAGCAGTTTCG  CTTCGTTACTGTCACTGCTTCCTC |
| A04_115 | A04_11994361 | A04_11545283 | GAAGGTGACCAAGTTCATGCTGGGTTTCATGAGGGTGGAGTTC  GAAGGTCGGAGTCAACGGATTGGGTTTCATGAGGGTGGAGTTG  AGACTGCTATTTCCGGACTTGATT |
| A04_131 | A04_13114842 | A04_15511956 | GAAGGTGACCAAGTTCATGCTAGTTCAAACACGTCGGATACAAAT  GAAGGTCGGAGTCAACGGATTAGTTCAAACACGTCGGATACAAAA  TGGTGGTGATTTCTCCCGATTTAT |
| A04_169 | A04_16938178 | A04_19386564 | GAAGGTGACCAAGTTCATGCTAGTAGGCATATCGTTCTAGAGTTTCT  GAAGGTCGGAGTCAACGGATTAGTAGGCATATCGTTCTAGAGTTTCG  TAGTCGGTAACCACGTTTACTTGT |
| A04_212 | A04_17841671 | A04_21217304 | GAAGGTGACCAAGTTCATGCTAAGCTGTTTTTCGGAAACTGTGTC  GAAGGTCGGAGTCAACGGATTAAGCTGTTTTTCGGAAACTGTGTT  TGAGGATGGTTCTTCTGATTCCAT |
| A04_230 | A04_19672559 | A04_23090434 | GAAGGTGACCAAGTTCATGCTTTTTGCTCGGGTCTGCAAAATACA  GAAGGTCGGAGTCAACGGATTTTTTGCTCGGGTCTGCAAAATACG  ACATTCTCTTTTCCAGACAAGTCA |
| A05_13 | A05_1332744 | A05_1300849 | GAAGGTGACCAAGTTCATGCTTCAAGGATGCAACTATTTGACTGT  GAAGGTCGGAGTCAACGGATTTCAAGGATGCAACTATTTGACTGC  AGGACAACGTATTAGGTTGGTCAT |
| A05_33 | A05_ 2735909 | A05_3388871 | GAAGGTGACCAAGTTCATGCTTGTAACGTGTGATCGATCTCATAT  GAAGGTCGGAGTCAACGGATTTGTAACGTGTGATCGATCTCATAC  GGACCCATACAAATAAATGGAACCA |
| A05_48 | A05_ 4877799 | A05_4863465 | GAAGGTGACCAAGTTCATGCTTCAGATTTTCGGGTCTCTATAAAATG  GAAGGTCGGAGTCAACGGATTTCAGATTTTCGGGTCTCTATAAAATA  TGCAATTTGGACTTTTCCCAAGTT |
| A05_116 | A05_ 9286467 | A05_11658481 | GAAGGTGACCAAGTTCATGCTTTGGACATCCTTGGTGAGAACATA GAAGGTCGGAGTCAACGGATTTTGGACATCCTTGGTGAGAACATC  CTCTAGTTCGCAAAGGTCCTACAA |
| A05_176 | A05_ 16771528 | A05_17611530 | GAAGGTGACCAAGTTCATGCTTCGGAAACCACAAATCTTGATCAAA GAAGGTCGGAGTCAACGGATTTCGGAAACCACAAATCTTGATCAAG  CAATATTAGGGATAGGACGGCTCC |
| A05_231 | A05_ 22399406 | A05_23126815 | GAAGGTGACCAAGTTCATGCTAAAAGTGCAAGAAAACGAAGTCAG  GAAGGTCGGAGTCAACGGATTAAAAGTGCAAGAAAACGAAGTCAC  TGTGATCATTTCTTCCTGTCCTCT |
| A05_266 | A05_ 23395814 | A05_26669944 | GAAGGTGACCAAGTTCATGCTGGTTCGACTTCGCGTACTCC  GAAGGTCGGAGTCAACGGATTGGTTCGACTTCGCGTACTCG  GAGGAGGAAAGTGTCTCTACCATC |
| A05_283 | A05_ 25113285 | A05_28380751 | GAAGGTGACCAAGTTCATGCTCCGGCCTGATTCAATTTCAAACC  GAAGGTCGGAGTCAACGGATTCCGGCCTGATTCAATTTCAAACT  GTCCTGCCGGAGTTTTCAAATC |
| A05_262 | A05_ 26262620 | A05_30067075 | GAAGGTGACCAAGTTCATGCTCACAAGCTAACAAACAGAATCAATTGT GAAGGTCGGAGTCAACGGATTCACAAGCTAACAAACAGAATCAATTGA CTACACACTTCATCGTCTCGAGAT |
| A05_318 | A05_ 27954728 | A05_31856933 | GAAGGTGACCAAGTTCATGCTATTGAGATCCGAGTTGCATCAGTA  GAAGGTCGGAGTCAACGGATTATTGAGATCCGAGTTGCATCAGTT CCGCTATAACTACTAGACTGCTGG |
| A06_22 | A06_ 2347833 | A06_2224830 | GAAGGTGACCAAGTTCATGCTAGCCACTCTGCAATGAAAGAGAAT  GAAGGTCGGAGTCAACGGATTAGCCACTCTGCAATGAAAGAGAAC  GACTGATACCTACTGCCCAATGTA |
| A06_31 | A06_ 3209268 | A06_3135921 | GAAGGTGACCAAGTTCATGCTTGTAAGAGTCATACACAAGATCGATATA  GAAGGTCGGAGTCAACGGATTTGTAAGAGTCATACACAAGATCGATATG  TCTTTTGATGGGATTGTAACTCTGT |
| A06_51 | A06_ 5223140 | A06_5144843 | GAAGGTGACCAAGTTCATGCTACACAGTTTAACTTGCTTCTCGTAC  GAAGGTCGGAGTCAACGGATTACACAGTTTAACTTGCTTCTCGTAG  CCGTTACATTTTCATCCACAACCA |
| A06_60 | A06_ 6618307 | A06_6049186 | GAAGGTGACCAAGTTCATGCTCTTACCACCTTCTGGGATTGTTG GAAGGTCGGAGTCAACGGATTCTTACCACCTTCTGGGATTGTTT  ATCTATTACGGGCTTTCCAGAGAG |
| A06_82 | A06_ 7654351 | A06_8299351 | GAAGGTGACCAAGTTCATGCTTCTCTCTGTGAAGCTGATAGTGAT  GAAGGTCGGAGTCAACGGATTTCTCTCTGTGAAGCTGATAGTGAA  GCTAGGCAAGTAGCTACACAAAGA |
| A06_101 | A06_ 9394768 | A06_10197929 | GAAGGTGACCAAGTTCATGCTGAATTGCAACCCAGTCTCTACTCC  GAAGGTCGGAGTCAACGGATTGAATTGCAACCCAGTCTCTACTCG  TCCATCTTCAGCAGAGAGTTTTGA |
| A06_160 | A06_ 16680187 | A06_16011759 | GAAGGTGACCAAGTTCATGCTTCCACATCATCAAAGAAATAGGGG  GAAGGTCGGAGTCAACGGATTTCCACATCATCAAAGAAATAGGGA  GAAAGCAGCTCGAGAAAGTTACAA |
| A06_170 | A06_ 17517003 | A06_17001540 | GAAGGTGACCAAGTTCATGCTAAACACACAACTTCACAACGAAAG  GAAGGTCGGAGTCAACGGATTAAACACACAACTTCACAACGAAAC  GATTCTTGATCATTCACGAGTGGG |
| A06_186 | A06_ 19042218 | A06_18629813 | GAAGGTGACCAAGTTCATGCTAAACCTCACACGCCTCCCAG  GAAGGTCGGAGTCAACGGATTAAACCTCACACGCCTCCCAC  AAAACGGAATCAGGAACCTATGGA |
| A06_207 | A06_ 20517836 | A06_20769324 | GAAGGTGACCAAGTTCATGCTGCATTTCTTTTTATGTTCCATTGAAAATATCAG  GAAGGTCGGAGTCAACGGATTGCATTTCTTTTTATGTTCCATTGAAAATATCAC  CGTATTCTTTGCCTAACTGGAAGT |
| A06_242 | A06_ 23932938 | A06_24235008 | GAAGGTGACCAAGTTCATGCTAGATAATTCGTTACTTTGTTTTCATGTT  GAAGGTCGGAGTCAACGGATTAGATAATTCGTTACTTTGTTTTCATGTA  CCAAACCATAACCAGAAGCAGAAA |
| A06_254 | A06_ 25150742 | A06_25416410 | GAAGGTGACCAAGTTCATGCTTTATATCCGAATATATACCTTGCGTATC  GAAGGTCGGAGTCAACGGATTTTATATCCGAATATATACCTTGCGTATT  GCAAGTCTCAGTCGGAAAAATCAA |
| A06_272 | A06_ 26423569 | A06_27277055 | GAAGGTGACCAAGTTCATGCTTGTTTCGACCTGTTTATTCTCTTTTTAATC  GAAGGTCGGAGTCAACGGATTTGTTTCGACCTGTTTATTCTCTTTTTAATG  TAAAATGGTGATTCAAACGGACCC |
| A06_303 | A06_ 28412694 | A06_30379566 | GAAGGTGACCAAGTTCATGCTTCTAAAGAAGACGGGATTTACTTGTC  GAAGGTCGGAGTCAACGGATTTCTAAAGAAGACGGGATTTACTTGTG  TCCCTTTTCTTCTGGTTTACCATT |
| A07_13 | A07_ 641639 | A07_1316624 | GAAGGTGACCAAGTTCATGCTAGCACCTTTTGTTTTTAGTTCTAAATC GAAGGTCGGAGTCAACGGATTAGCACCTTTTGTTTTTAGTTCTAAATG  TGCAGCAGAGCTAACTAACTGATA |
| A07_37 | A07_ 3093329 | A07_3759833 | GAAGGTGACCAAGTTCATGCTTGGAGCAGAAAGTGTCAAATTGTAT  GAAGGTCGGAGTCAACGGATTTGGAGCAGAAAGTGTCAAATTGTAG  GAGATCCGAAATACCTGATCCGAA |
| A07_119 | A07_ 9886537 | A07_11953345 | GAAGGTGACCAAGTTCATGCTTGTCACTCTTTTGCCATCTATGATTTT  GAAGGTCGGAGTCAACGGATTTGTCACTCTTTTGCCATCTATGATTTC  ACATACTTCCATGGCAAAACAAGA |
| A07_133 | A07_ 11216548 | A07_13327175 | GAAGGTGACCAAGTTCATGCTTGAAGGTTTGGATATATATTAATCTCAATCT  GAAGGTCGGAGTCAACGGATTTGAAGGTTTGGATATATATTAATCTCAATCC  ACCAGATTCTGATGGGATGGAAAT |
| A07_142 | A07_ 11504569 | A07_14299498 | GAAGGTGACCAAGTTCATGCTGCTTCGAATTGAGGAGTCGTTC  GAAGGTCGGAGTCAACGGATTGCTTCGAATTGAGGAGTCGTTG  TCTCAGCATTACGATCATCACTCT |
| A07_158 | A07_ 13063095 | A07_15858440 | GAAGGTGACCAAGTTCATGCTAGAGGAATAAGAAGCTATAAAAACAGG  GAAGGTCGGAGTCAACGGATTAGAGGAATAAGAAGCTATAAAAACAGA  AGAAAATATGCTTTGAATGGGAATCA |
| A07_173 | A07_ 14530553 | A07_17380939 | GAAGGTGACCAAGTTCATGCTCTAGATAAGCCAAGACTGCGTGCT  GAAGGTCGGAGTCAACGGATTCTAGATAAGCCAAGACTGCGTGCC  TTGTCTGCCGTTTGTTATAAGAGC |
| A07_198 | A07_ 16897498 | A07_19833366 | GAAGGTGACCAAGTTCATGCTTTTGTTGGCGTATATAGGAAGAAC GAAGGTCGGAGTCAACGGATTTTTGTTGGCGTATATAGGAAGAAG  ACAAAAGCAACACACTTGTAGGAC |
| A07_207 | A07_ 17388245 | A07_20721940 | GAAGGTGACCAAGTTCATGCTACATACAATAACATCGATCTTGGAACT  GAAGGTCGGAGTCAACGGATTACATACAATAACATCGATCTTGGAACC  ATGGTCTGAGGATGTTAACTTGCT |
| A07_245 | A07_ 19099777 | A07_24502213 | GAAGGTGACCAAGTTCATGCTCTTGTCTTCCCATGGTCACTCCT  GAAGGTCGGAGTCAACGGATTCTTGTCTTCCCATGGTCACTCCA  CAGTTCACGGGCTTTAACTTTCAG |
| A07_260 | A07_ 20870143 | A07_26014154 | GAAGGTGACCAAGTTCATGCTGCCTTTGTCTTTTGAAATTTCCAACT  GAAGGTCGGAGTCAACGGATTGCCTTTGTCTTTTGAAATTTCCAACA  CACCCACCACTAAGTAACAACCTA |
| A07_277 | A07_ 22341484 | A07_27759083 | GAAGGTGACCAAGTTCATGCTCGTATCTATGGAGATTTTCATGTGACG  GAAGGTCGGAGTCAACGGATTCGTATCTATGGAGATTTTCATGTGACT  GACATATCTCTCTGAGGCTCGCTA |
| A07_290 | A07_ 23495468 | A07_29084853 | GAAGGTGACCAAGTTCATGCTTTCAATTCGGTAAGACGTGCTCAT  GAAGGTCGGAGTCAACGGATTTTCAATTCGGTAAGACGTGCTCAG  TAAGCATCCCAAATGAGTTTGTGG |
| A07_306 | A07_ 24904486 | A07_30642246 | GAAGGTGACCAAGTTCATGCTCAAGTTGATCGGTTTCACTAAGGG  GAAGGTCGGAGTCAACGGATTCAAGTTGATCGGTTTCACTAAGGA  CTGGGCGAGTCAGACATTTTATTC |
| A07_327 | A07_ 26714270 | A07_32737350 | GAAGGTGACCAAGTTCATGCTAGCTGCATTGTATTCTCCAAGTTT  GAAGGTCGGAGTCAACGGATTAGCTGCATTGTATTCTCCAAGTTC  TCAGTGCGTGGTTTCTTTCAAATT |
| A08_48 | A08_4234497 | A08_4825591 | GAAGGTGACCAAGTTCATGCTCACCAAGTTAGAAACAACGACAAC GAAGGTCGGAGTCAACGGATTCACCAAGTTAGAAACAACGACAAA  CATGCTATGGTGGGGATCTTTTTC |
| A08_99 | A08_ 7817882 | A08_9944344 | GAAGGTGACCAAGTTCATGCTTCAAGAATGATAGACAAGGCAGCA  GAAGGTCGGAGTCAACGGATTTCAAGAATGATAGACAAGGCAGCG  GGTGAGTTTCAATTCCTTGCTGTT |
| A08_138 | A08_ 11662041 | A08_13870242 | GAAGGTGACCAAGTTCATGCTTTTGAAGACCAATCTTTTGAGCAT  GAAGGTCGGAGTCAACGGATTTTTGAAGACCAATCTTTTGAGCAC  TAACTCTACACTTTTTCGCTTCGC |
| A08_156 | A08_ 2608691 | A08_ 15666303 | GAAGGTGACCAAGTTCATGCTCGAGAATCTCCCTCGCGTTA  GAAGGTCGGAGTCAACGGATTCGAGAATCTCCCTCGCGTTG  GGATTGGCTAGAGATAGCGAGAAG |
| A08_180 | A08_ 14790903 | A08_18023255 | GAAGGTGACCAAGTTCATGCTCCAAGTCATAGTGTTATTAATTAGTAGGTTTCT  GAAGGTCGGAGTCAACGGATTCCAAGTCATAGTGTTATTAATTAGTAGGTTTCA  TTCCCTTGCTAAAAAGTCGGAGTA |
| A08_195 | A08_ 16292255 | A08_19541532 | GAAGGTGACCAAGTTCATGCTACAAACTACATAATAAAGTTCTACAACGA GAAGGTCGGAGTCAACGGATTACAAACTACATAATAAAGTTCTACAACGG  TAGCCCTCCTATGAAAATTCACGT |
| A08_207 | A08_ 17655268 | A08_20772381 | GAAGGTGACCAAGTTCATGCTGCAAGAACTTGAGAGATGAAAGAGATA  GAAGGTCGGAGTCAACGGATTGCAAGAACTTGAGAGATGAAAGAGATG  GATACGTTCGTTATGCATATGGGC |
| A08_215 | A08_ 18470828 | A08_21587866 | GAAGGTGACCAAGTTCATGCTACCATAATAGCCTCGTCTAGTAACG GAAGGTCGGAGTCAACGGATTACCATAATAGCCTCGTCTAGTAACA GCTGTGAGAACATCATTGGTGAAT |
| A08_245 | A08_ 21234115 | A08_24528415 | GAAGGTGACCAAGTTCATGCTCTGTTGTTCTCGGTGGATCTCAA  GAAGGTCGGAGTCAACGGATTCTGTTGTTCTCGGTGGATCTCAC  GAAAAACTCAGAAGCATTCACGGA |
| A08_250 | A08_ 22051734 | A08_25015222 | GAAGGTGACCAAGTTCATGCTCCCCTTTTAATAAATAACATATTGGGCCTA  GAAGGTCGGAGTCAACGGATTCCCCTTTTAATAAATAACATATTGGGCCTG  AAGGGCCTTATAATGTGGGTTCTT |
| A09_34 | A09_ 1993503 | A09_3400590 | GAAGGTGACCAAGTTCATGCTCCCATTCTGATGTATGTAAGCACAC  GAAGGTCGGAGTCAACGGATTCCCATTCTGATGTATGTAAGCACAT  CTACTATCCGGCTCCTGATTTCAA |
| A09_51 | A09_ 3652415 | A09_5153459 | GAAGGTGACCAAGTTCATGCTACGCATAATTCAGCCATTTAATATTTC GAAGGTCGGAGTCAACGGATTACGCATAATTCAGCCATTTAATATTTT TAGATCATCTGGGCTCTAGTCAGT |
| A09_69 | A09_ 5879670 | A09_6994064 | GAAGGTGACCAAGTTCATGCTAGACTGACTATCGTGTTAGGTTCC  GAAGGTCGGAGTCAACGGATTAGACTGACTATCGTGTTAGGTTCA  GACAAAGAAGTAGTTGAAGCACGT |
| A09_138 | A09_ 10345925 | A09_13882727 | GAAGGTGACCAAGTTCATGCTATCAAGATATAAAGATCAGAGGAGAATTAG  GAAGGTCGGAGTCAACGGATTATCAAGATATAAAGATCAGAGGAGAATTAC  AGCAACGGTCATGAACAAATAAGG |
| A09_163 | A09_ 12625905 | A09_16389444 | GAAGGTGACCAAGTTCATGCTATCTTTGACTTAATCGTCGTTGGG  GAAGGTCGGAGTCAACGGATTATCTTTGACTTAATCGTCGTTGGT  ATGATTTCACAGAACAAACCCACC |
| A09_193 | A09_ 14910436 | A09_19340010 | GAAGGTGACCAAGTTCATGCTTTTTATAATAAACGCCATTCCGCC  GAAGGTCGGAGTCAACGGATTTTTTATAATAAACGCCATTCCGCG  TTTCGATGGGAAAAGCAAACACAC |
| A09_204 | A09_ 16001832 | A09_20464228 | GAAGGTGACCAAGTTCATGCTGTTGCGGTCAGATCAGCCC  GAAGGTCGGAGTCAACGGATTGTTGCGGTCAGATCAGCCG  AGTCACCGAAGAACTCACATGAAT |
| A09_279 | A09_ 24490657 | A09_27965809 | GAAGGTGACCAAGTTCATGCTGTAGGAACTAGGAAAGCTGGTTTTT  GAAGGTCGGAGTCAACGGATTGTAGGAACTAGGAAAGCTGGTTTTG  CCATGAGAGCAGAAGAAACATCAC |
| A09_303 | A09_ 26582428 | A09_30301982 | GAAGGTGACCAAGTTCATGCTATCCCCACATAGTCTTGGAAAAAT  GAAGGTCGGAGTCAACGGATTATCCCCACATAGTCTTGGAAAAAC  TCACTTCACTTGGTAGAGGAAAGG |
| A09_339 | A09_ 29144941 | A09_33937386 | GAAGGTGACCAAGTTCATGCTTGATGTGTCTAGATGAGTCTCTTTCC  GAAGGTCGGAGTCAACGGATTTGATGTGTCTAGATGAGTCTCTTTCT  AAGGAATTAGACCTTGAATTCAATCA |
| A09_416 | A09_ 33100083 | A09_41614373 | GAAGGTGACCAAGTTCATGCTAGCATTTATTTGGTTTTGTGTCTTCTA  GAAGGTCGGAGTCAACGGATTAGCATTTATTTGGTTTTGTGTCTTCTG  GATAAGGAGAGAGAGATTACCCGC |
| A09_438 | A09_ 34682022 | A09_43840445 | GAAGGTGACCAAGTTCATGCTTGGTTTACCATTCTAGATTTCACTG  GAAGGTCGGAGTCAACGGATTTGGTTTACCATTCTAGATTTCACTA  CTCTGGATCCAGTAGTACTGTTGG |
| A09_464 | A09_ 37114820 | A09_46429597 | GAAGGTGACCAAGTTCATGCTTCTCTTTCCTCTCCATTTTTACATAATTG  GAAGGTCGGAGTCAACGGATTTCTCTTTCCTCTCCATTTTTACATAATTT  AGGGATTATGGTAACGCACTGTTA |
| A09_503 | A09_ 40445161 | A09_50327852 | GAAGGTGACCAAGTTCATGCTAGAGAGATACGAATCGAAAACGGT GAAGGTCGGAGTCAACGGATTAGAGAGATACGAATCGAAAACGGC  TTTATTTACGCCGAACTTCCCAAC |
| A09_526 | A09_ 42388741 | A09_52632495 | GAAGGTGACCAAGTTCATGCTTCAGAGGTGACGGTATAGGAGAAA  GAAGGTCGGAGTCAACGGATTTCAGAGGTGACGGTATAGGAGAAC  CGAGCCTAGATTCTCTTTCTCCTC |
| A10_30 | A10_ 1104038 | A10_3091413 | GAAGGTGACCAAGTTCATGCTCGGTTTAGTTCCTTTCCTGATCATG  GAAGGTCGGAGTCAACGGATTCGGTTTAGTTCCTTTCCTGATCATA  GTATAGTTTAGTGATCGGTGGGCT |
| A10_34 | A10_ 749385 | A10_3453520 | GAAGGTGACCAAGTTCATGCTCAATGAACATGACAACGGTTTCG  GAAGGTCGGAGTCAACGGATTCAATGAACATGACAACGGTTTCA  AGTTGAATCCACCTGTAGAAGCAT |
| A10_50 | A10_ 1340015 | A10_5000546 | GAAGGTGACCAAGTTCATGCTCCGAGTCAGAAGCCAAAACTCTA  GAAGGTCGGAGTCAACGGATTCCGAGTCAGAAGCCAAAACTCTC  GTCAGGTCTTACTGCAACAACTTC |
| A10_67 | A10_ 8565096 | A10_6760754 | GAAGGTGACCAAGTTCATGCTCAGTACCGGCCTCAATGAAAC  GAAGGTCGGAGTCAACGGATTCAGTACCGGCCTCAATGAAAA  ACTTGTTAGTCCTGTTCCTTTGGT |
| A10_76 | A10_ 9393250 | A10_7599922 | GAAGGTGACCAAGTTCATGCTTGATTGCGGTCTTTCTTTCCAAAGT  GAAGGTCGGAGTCAACGGATTTGATTGCGGTCTTTCTTTCCAAAGC  TCGATAGACCCCTGAATCATGTTT |
| A10_86 | A10_ 10075492 | A10_8693019 | GAAGGTGACCAAGTTCATGCTAGCTTTTACATACTTTAAGTAGTCCACT  GAAGGTCGGAGTCAACGGATTAGCTTTTACATACTTTAAGTAGTCCACA  GCTTTTAAGTGTATCGTCCGTGTT |
| A10_114 | A10_ 13060913 | A10_11484992 | GAAGGTGACCAAGTTCATGCTTGTTACTTGTTTGGTTGTTGACATAGTA  GAAGGTCGGAGTCAACGGATTTGTTACTTGTTTGGTTGTTGACATAGTT  TGCATCTTCTCAACCTCTTCTCAT |
| A10_115 | A10_ 13145055 | A10_11564208 | GAAGGTGACCAAGTTCATGCTGGTAATTCATGTTGTGGTAAATGGTTC  GAAGGTCGGAGTCAACGGATTGGTAATTCATGTTGTGGTAAATGGTTA  GAGGATAGCAAACATGCTGACTTG |
| A10_128 | A10_ 14520072 | A10_12897525 | GAAGGTGACCAAGTTCATGCTGGATATCTATCACACGTCGCACTC  GAAGGTCGGAGTCAACGGATTGGATATCTATCACACGTCGCACTT  ACTTCCTTGATGTTATCATTCATATGCA |
| A10_146 | A10_ 16255485 | A10_14668421 | GAAGGTGACCAAGTTCATGCTACGTACACCACTGAAAGATAATCTT  GAAGGTCGGAGTCAACGGATTACGTACACCACTGAAAGATAATCTC  TGACTATGACCGGAAACAAGTTTT |
| A10_155 | A10_ 17032719 | A10_15589544 | GAAGGTGACCAAGTTCATGCTCTAGCAACCTTCGTCATAGCCC  GAAGGTCGGAGTCAACGGATTCTAGCAACCTTCGTCATAGCCG  AACGTAGAGGAGAAGCAACTGATG |
| A10_163 | A10_ 17699757 | A10_16377540 | GAAGGTGACCAAGTTCATGCTTGTAAATATGGCTTATGAAGTTATGTCG  GAAGGTCGGAGTCAACGGATTTGTAAATATGGCTTATGAAGTTATGTCT  GGCAAGAAGACAACAGAACCATAG |
| A10_180 | A10_ 18668656 | A10_18005152 | GAAGGTGACCAAGTTCATGCTATGAGCGTGGGGAAAGCGA  GAAGGTCGGAGTCAACGGATTATGAGCGTGGGGAAAGCGG  GAAGCTCCCTTCTCCTTCATATCA |
| A10_188 | A10_ 20005102 | A10_18867232 | GAAGGTGACCAAGTTCATGCTTCATAAACCTGCATGCATCCAATC  GAAGGTCGGAGTCAACGGATTTCATAAACCTGCATGCATCCAATT  TCGAAGAAAAGTTCATAACAAGAGAA |

Supplementary Table 3 The average genetic distance among different types of investigated materials

| Average genetic distance | Heading mustard | Root mustard | Potherb mustard | Introgression  type Ⅰ | Introgression  type IV | Introgression  type IV | Chinese cabbage |
| --- | --- | --- | --- | --- | --- | --- | --- |
| Heading mustard | 0.02 | 0.34 | 0.26 | 0.37 | 0.22 | 0.34 | 0.53 |
| Root mustard | 0.34 | 0.16 | 0.32 | 0.39 | 0.39 | 0.39 | 0.44 |
| Potherb mustard | 0.26 | 0.32 | 0.19 | 0.35 | 0.35 | 0.34 | 0.48 |
| Introgression  type I | 0.37 | 0.39 | 0.35 | 0.20 | 0.31 | 0.30 | 0.47 |
| Introgression  type IV | 0.22 | 0.39 | 0.35 | 0.31 | 0.20 | 0.28 | 0.47 |
| Introgression  type V | 0.34 | 0.39 | 0.34 | 0.30 | 0.28 | 0.17 | 0.45 |
| Chinese cabbage | 0.53 | 0.44 | 0.48 | 0.47 | 0.47 | 0.45 | 0.21 |

Supplementary Table 4 The maximum genetic distance among different types of investigated materials

| Maximum genetic distance | Heading mustard | Root mustard | Potherb mustard | Introgression  type Ⅰ | Introgression  type IV | Introgression  type V | Chinese cabbage |
| --- | --- | --- | --- | --- | --- | --- | --- |
| Heading mustard | 0.03 | 0.38 | 0.29 | 0.43 | 0.34 | 0.43 | 0.56 |
| Root mustard | 0.38 | 0.25 | 0.38 | 0.43 | 0.42 | 0.42 | 0.50 |
| Potherb mustard | 0.29 | 0.38 | 0.25 | 0.41 | 0.41 | 0.41 | 0.52 |
| Introgression  type I | 0.43 | 0.43 | 0.41 | 0.33 | 0.42 | 0.39 | 0.53 |
| Introgression  type IV | 0.34 | 0.42 | 0.41 | 0.42 | 0.31 | 0.38 | 0.53 |
| Introgression  type V | 0.43 | 0.42 | 0.41 | 0.39 | 0.38 | 0.30 | 0.51 |
| Chinese cabbage | 0.56 | 0.50 | 0.52 | 0.53 | 0.53 | 0.51 | 0.28 |

**Figures**


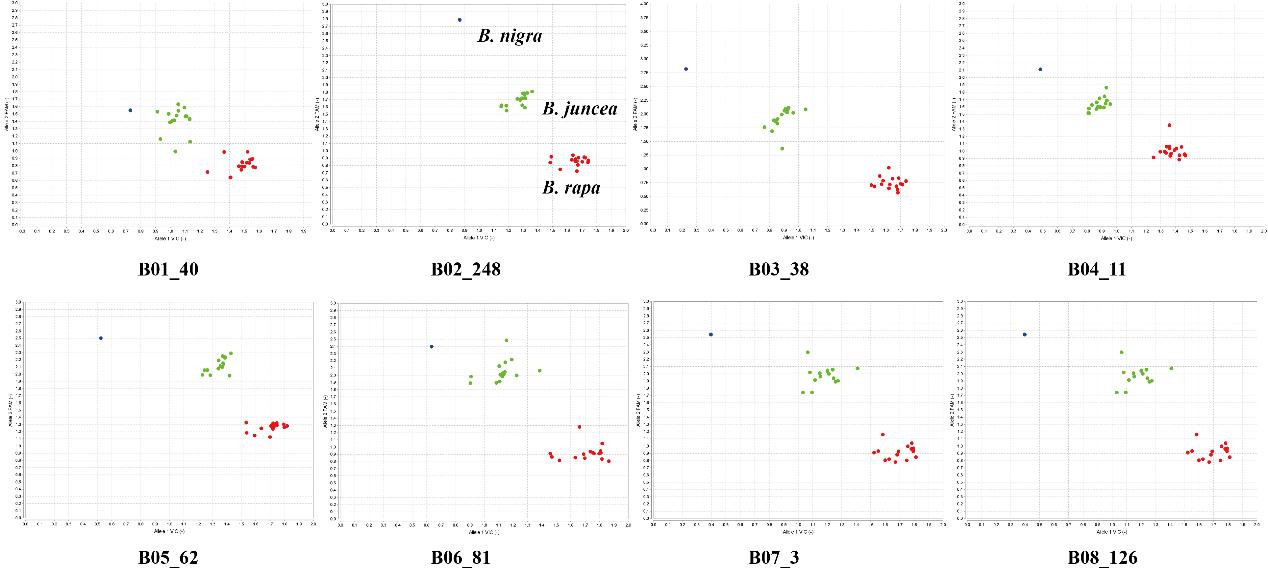


Supplementary Fig. 1 KASP genotyping of B genome markers in 16 *B. rapa* accessions and 16 *B. juncea* accessions and a *B. nigra* accession


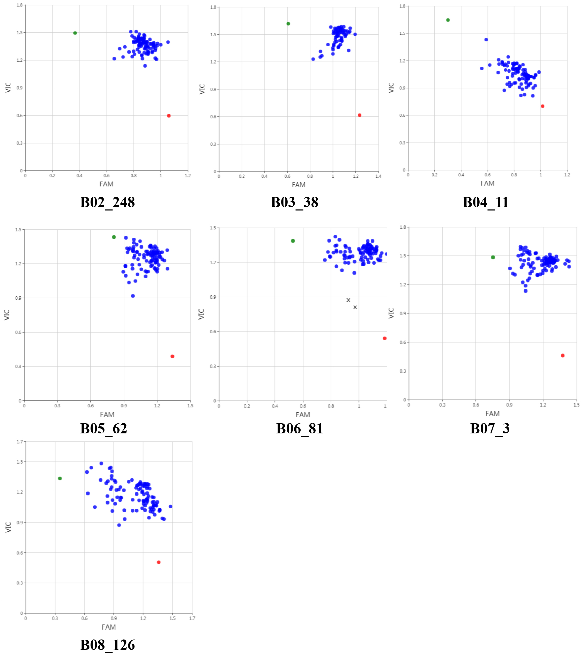


Supplementary Fig. 2 Genotyping for KASP markers on chromosomes B02 - B07. The blue dot represents the *B. juncea* genotype


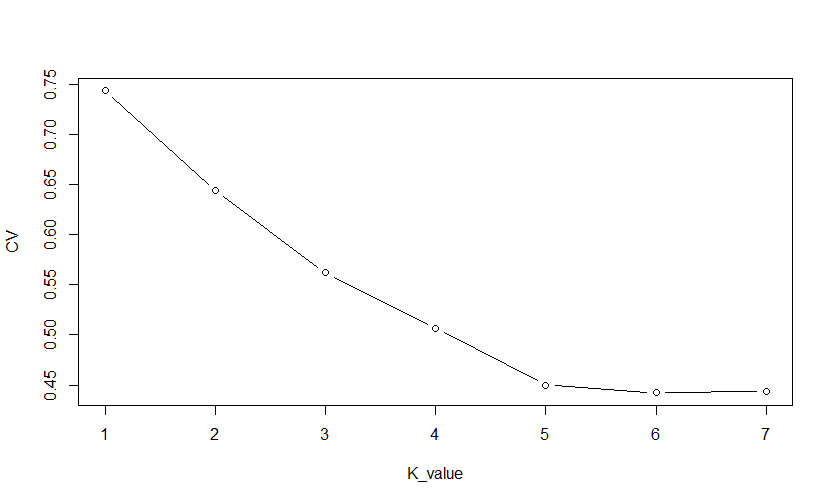


Supplementary Fig. 3 K values and CV values based on the ADMIXTURE program


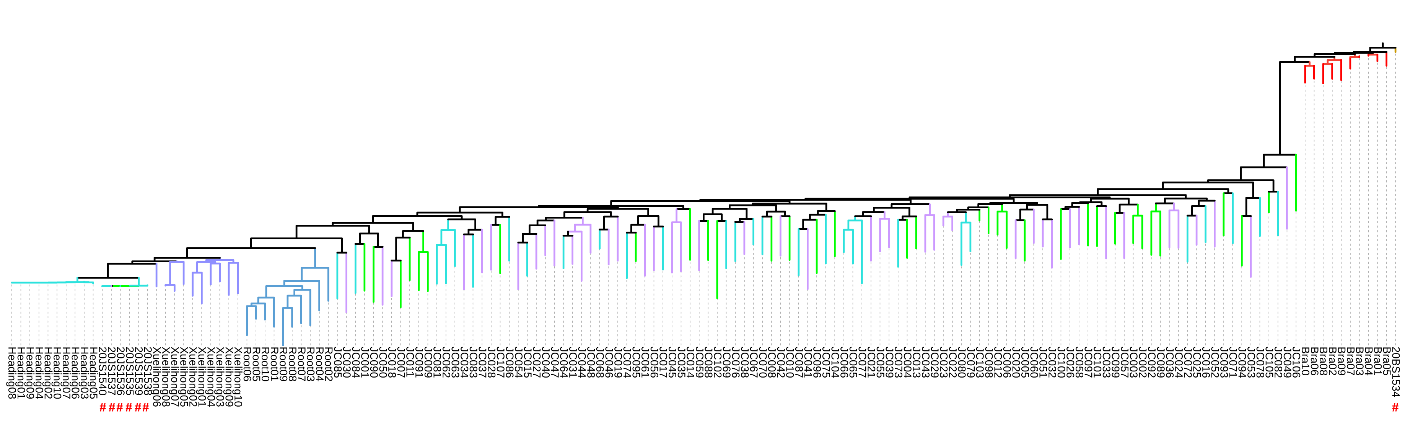


Supplementary Fig. 4 Phylogenetic tree of 154 individuals based on 132 SNPs. The red # represents the parental materials


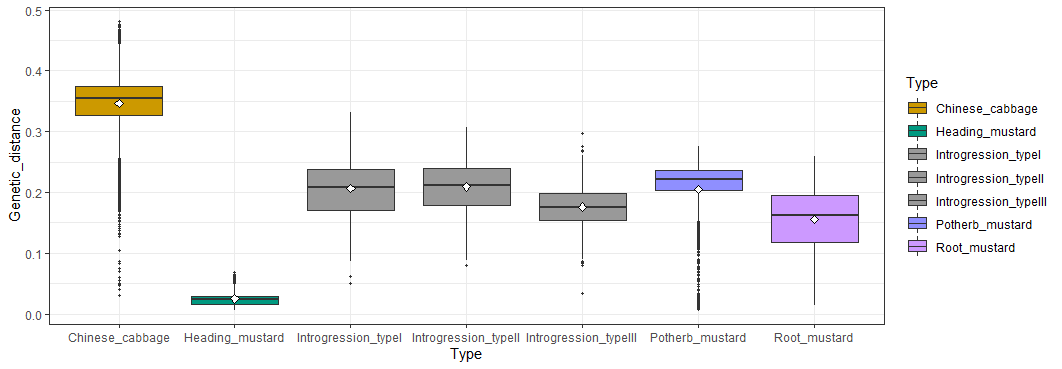


Supplementary Fig. 5 Genetic distance calculated after adding 188 *B. rapa* and 110 *B. juncea* accessions based on 132 SNPs
